# Updates on mouse models of Alzheimer’s disease

**DOI:** 10.1186/s13024-024-00712-0

**Published:** 2024-03-11

**Authors:** Michael Z. Zhong, Thomas Peng, Mariana Lemos Duarte, Minghui Wang, Dongming Cai

**Affiliations:** 1 Department of Neurology, Icahn School of Medicine at Mount Sinai, New York, NY 10029 USA; 2 Department of Genetics and Genomic Sciences, Icahn School of Medicine at Mount Sinai, New York, NY 10029 USA; 3 Department of Biology, College of Arts and Science, Boston University, Boston, MA 02215 USA; 4 Science Research Program, Scarsdale High School, New York, NY 10583 USA; 5 Research & Development, James J Peters VA Medical Center, Bronx, NY 10468 USA; 6 Icahn Institute for Data Science and Genomic Technology, Icahn School of Medicine at Mount Sinai, One Gustave L. Levy Place, New York, NY 10029 USA; 7 Mount Sinai Center for Transformative Disease Modeling, Icahn School of Medicine at Mount Sinai, One Gustave L. Levy Place, New York, NY 10029 USA; 8 Alzheimer’s Disease Research Center, Icahn School of Medicine at Mount Sinai, New York, NY 10029 USA; 9 Ronald M. Loeb Center for Alzheimer’s Disease, Icahn School of Medicine at Mount Sinai, New York, NY 10029 USA; 10 Department of Neurology, N. Bud Grossman Center for Memory Research and Care, The University of Minnesota, Minneapolis, MN 55455 USA; 11 Geriatric Research Education & Clinical Center (GRECC), The Minneapolis VA Health Care System, Minneapolis, MN 55417 USA

## Abstract

Alzheimer’s disease (AD) is the most common neurodegenerative disease in the United States (US). Animal models, specifically mouse models have been developed to better elucidate disease mechanisms and test therapeutic strategies for AD. A large portion of effort in the field was focused on developing transgenic (Tg) mouse models through over-expression of genetic mutations associated with familial AD (FAD) patients. Newer generations of mouse models through knock-in (KI)/knock-out (KO) or CRISPR gene editing technologies, have been developed for both familial and sporadic AD risk genes with the hope to more accurately model proteinopathies without over-expression of human AD genes in mouse brains. In this review, we summarized the phenotypes of a few commonly used as well as newly developed mouse models in translational research laboratories including the presence or absence of key pathological features of AD such as amyloid and tau pathology, synaptic and neuronal degeneration as well as cognitive and behavior deficits. In addition, advantages and limitations of these AD mouse models have been elaborated along with discussions of any sex-specific features. More importantly, the omics data from available AD mouse models have been analyzed to categorize molecular signatures of each model reminiscent of human AD brain changes, with the hope to guide future selection of most suitable models for specific research questions to be addressed in the AD field.

**Supplementary Information:**

The online version contains supplementary material available at 10.1186/s13024-024-00712-0.

## Introduction

Alzheimer’s disease (AD) is the most common neurodegenerative disease in the United States (US). Often found in elderly patients, aged 65 and above, it is characterized by memory loss, confusion, and behavioral changes [1]. It is also the 6th leading cause of death in the US and the leading cause of death in elderly. In 2021, the healthcare costs for AD patients in the US are about $355 billion and expected to be increased to $1.1 trillion in 2050 [2]. Despite the encouraging results from recent clinical trials of anti-amyloid antibody therapies [3, 4], the modest beneficial effects at slowing the rate of cognitive decline with significant side-effects indicate the urgent needs of developing more efficacious disease modifying therapies for AD.

The cardinal features of AD pathology are amyloid plaques due to excessive Aβ accumulation and neurofibrillary tangles (NFT) due to tau hyperphosphorylation [5]. These changes lead to neuronal death and cortical volume loss [1]. Besides autosomal dominant mutations of three genes (*APP*, *PSEN1* and *PSEN2*) identified in familial AD patients, there are risk factors associated with late-onset AD (LOAD) such as Apolipoprotein E4 (*APOE4*) and Triggering Receptor Expressed on Myeloid Cells 2 (*TREM2*). The *APOE4* allele is the strongest risk factor associated with AD. Conversely the *APOE2* allele is the strongest genetic protective factor against AD [6]. *TREM2* is a receptor involved in the function of microglia in the CNS and variants of *TREM2* have been associated with an increased risk of developing AD. It is suggested that TREM2 is vital in the microglial role in phagocytosis of cellular debris like Aβ [7]. Animal models, specifically mouse models carrying various AD risk genes such as *APOE4* and *TREM2* variants, have been developed to better elucidate disease mechanisms and test therapeutic strategies.

Generally, animal models aim to display the key pathological features of AD such as amyloid and tau pathology, as well as synaptic and neuronal degeneration. In addition, AD pathology should be developed in areas of the brain in a predictable way, similarly to disease progression in human AD patients. More importantly, cognitive function deterioration should follow human disease trajectory. A large portion of effort has been focused on developing transgenic (Tg) mouse models through over-expression of genetic mutations associated with familial AD (FAD) patients [8]. While some AD pathology such as amyloid plaques, neuroinflammation and cognitive impairment can be captured in these mouse models, many fail to display significant degeneration and neuronal loss. Newer generations of mouse models through knock-in (KI)/knock-out (KO) or Clustered Regularly Interspaced Short Palindromic Repeats (CRISPR) gene editing technologies, have been developed with the hope to more accurately model proteinopathies without over-expression of human AD genes in mouse brains.

**Fig. 1:**
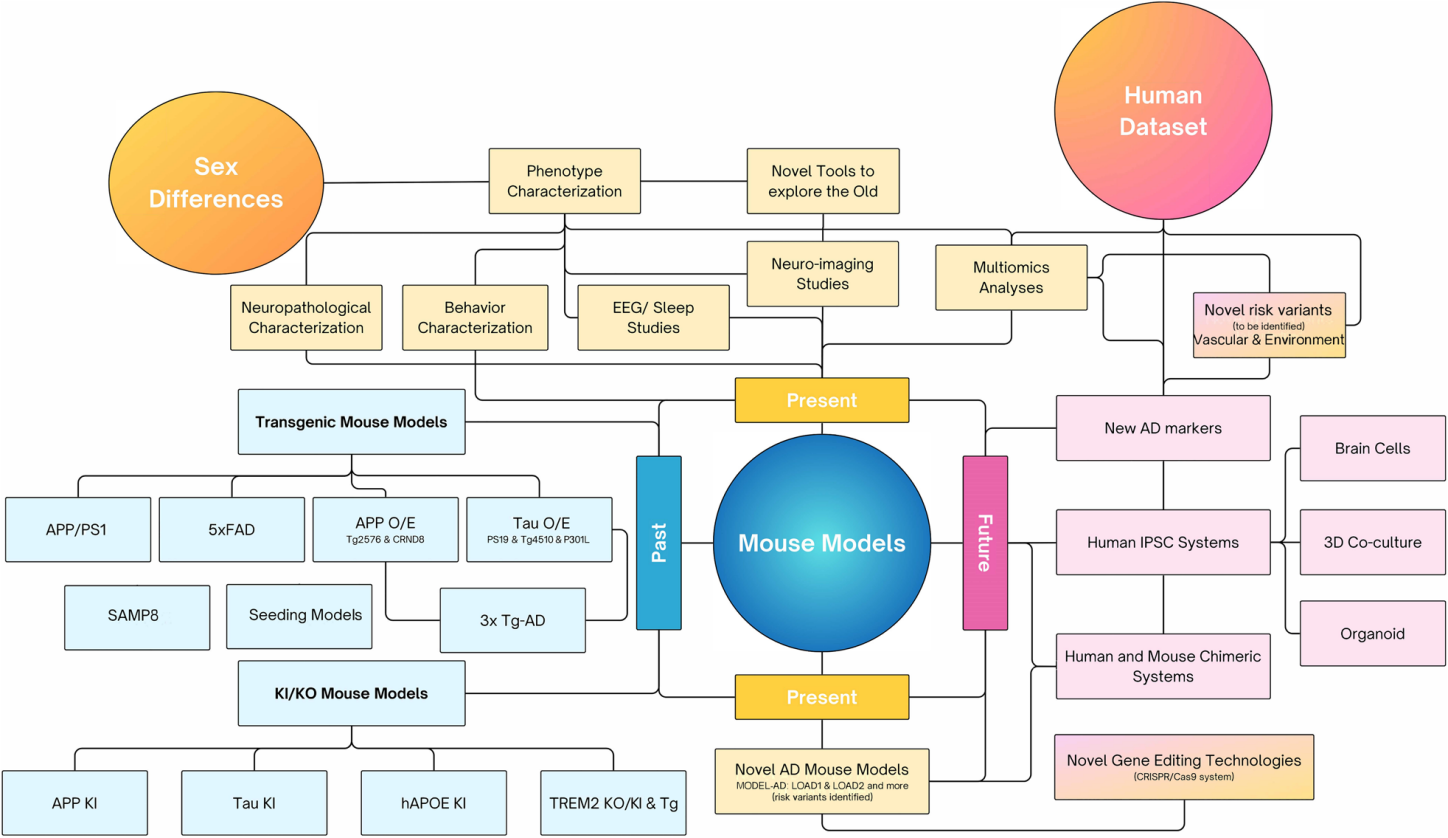
Summary of past, present and future in vivo model studies of AD. Traditional mouse models used in the past included various transgenic and KI/KO mouse models with phenotypic characterization of neuropathological and behavior changes. With new information gathered about novel risk variants of AD as well as new AD biomarkers, novel tools have been developed to deep phenotype many of existing mouse models of AD including EEG/sleep studies, neuro-imaging modalities as well as multi-omics analyses. The integration of mouse and human datasets facilitate a better understanding of molecular signatures of each mouse model reminiscent of human AD brain molecular signatures. Future directions such as developing new mouse models carrying novel AD risk variants to be identified, as well as human and mouse chimeric system to incorporate human iPSC systems into mouse models through transplantation approaches may provide novel insights into future in vivo modeling of AD

**Fig. 2:**
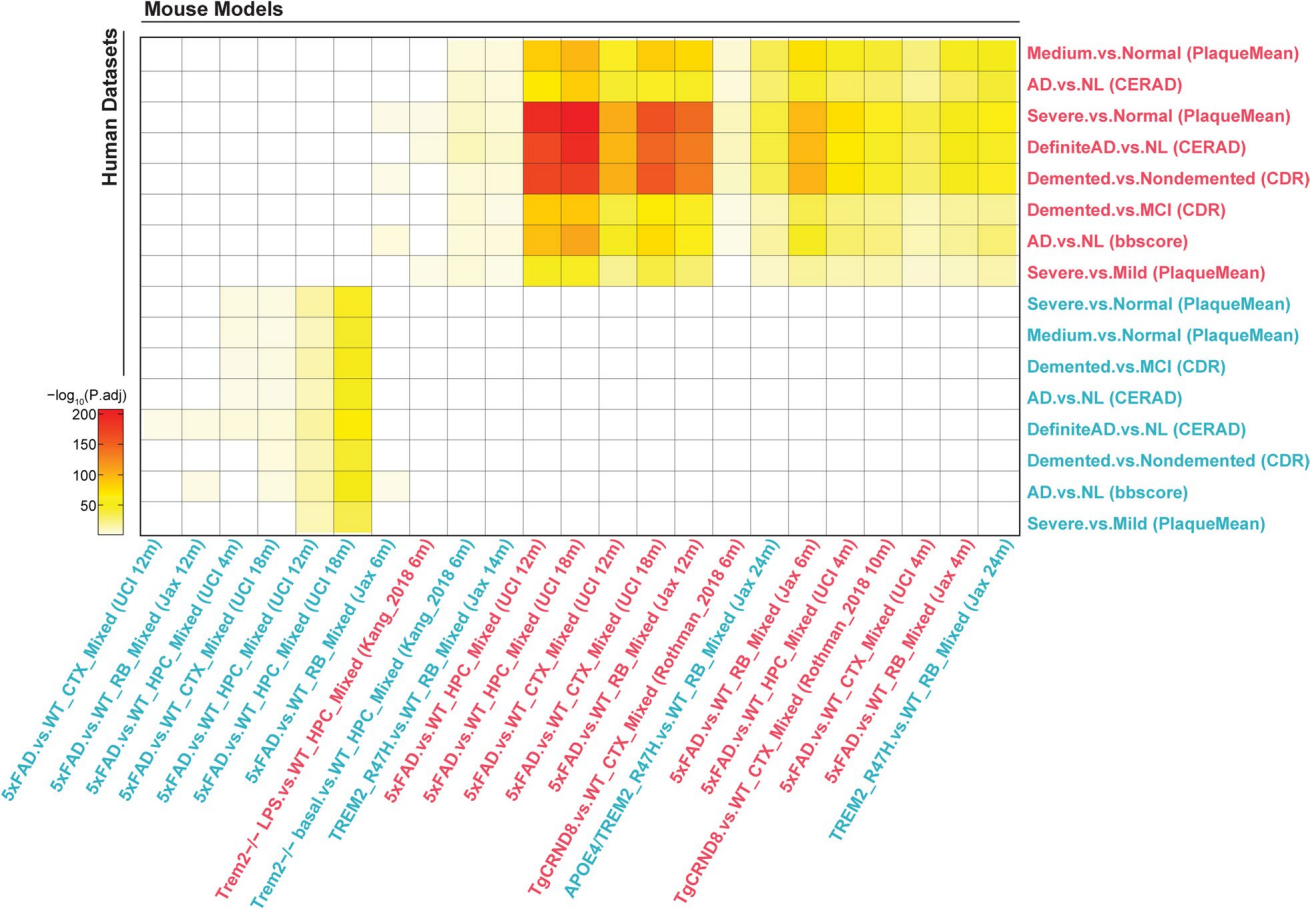
Comparison of molecular gene signatures between AD mouse model datasets and human AD brain datasets. Heatmap shows the *p* value significance (-Log_10_adjusted *p* values) of the overlaps between AD mouse model molecular signatures and human brain molecular signatures in relate to multiple cognitive/pathological traits (plaque means, CDR, Braak score, CERAD) derived from the PHG brain regions of the Mount Sinai Brain Bank (MSBB) cohort. Axis labels denote the trait contrasts for each gene signature overlap between mouse and human studies. Down- and up-regulated gene signatures are labeled in blue and red colors, respectively

**Fig. 3:**
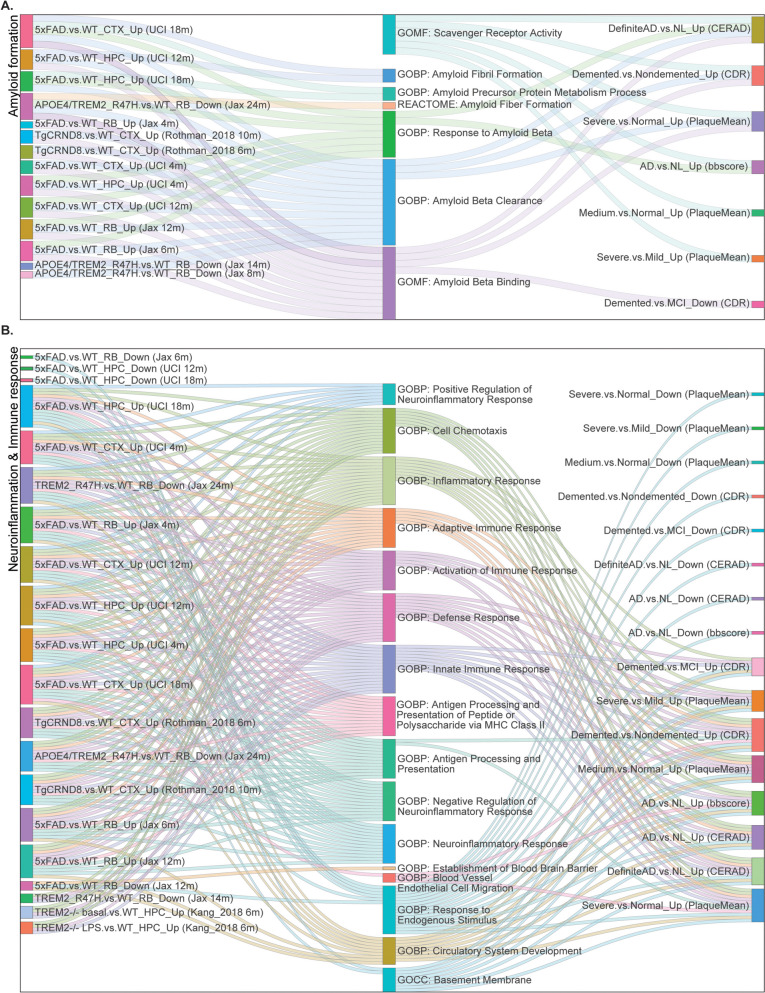
Comparison of gene ontology (GO)/pathways between the AD mouse model datasets and human AD brain datasets. Sanky network plots show the commonly shared GO/pathways between mouse (left) and human (right) gene signatures. Each node represents a gene signature or a GO/pathway term. Each link colored based of individual GO/pathway term represents a significant overlap between mouse and human gene signatures. **A** Commonly shared GO/pathways involved in amyloid processes in AD; **B** Commonly shared GO/pathways involved in neuroinflammation and immune responses in AD

In this review, we summarized a few well-established and commonly used mouse models, as well as newly developed mouse models of AD developed in translational research laboratories today, including both the traditional Tg mouse models, the new KI/KO models and other mouse models such as SAMP8 and seeding models (Fig. 1). For Tg mouse models, we included Tg2576, TgCRND8, APP/PS1, 5xFAD, and 3xTg-AD. We also discussed tauopathy models such as P301S, rTg4510 and P301L. For non-transgenic mouse models, APP-KI, Tau-KI, human APOE KI, TREM2-KO, hTREM2 KI and TREM2 Tg mouse models were discussed. The advantages and limitations of some of these AD mouse models have been recently discussed [9]. In this review, we further compared newly developed AD mouse models (e.g. TREM2 KO/KI and Tg mouse models, the MODEL-AD consortium LOAD mouse models, SAMP8 and seeding models) with previously well-established mouse models, including phenotypic characterization along with discussions of any sex-specific features (as summarized in Tables 1 and 2). More importantly, the publicly available transcriptomics data of various AD mouse models have been analyzed to categorize molecular signatures of each mouse model reminiscent of human AD brain changes (Figs. 2 and 3). Our proof-of-concept analyses compare phenotypic characterization with molecular signatures of mouse models in alignment with human AD brain signature changes, with the hope to guide our future effort to better characterize AD molecular and phenotypic signatures in new-generation mouse models and more importantly, to direct our selection of best mouse models for specific research questions to be addressed in the field. Finally, future needs of developing novel model systems for AD have been discussed such as developing new mouse models carrying novel AD risk variants to be identified or novel model systems to capture heterogenous disease mechanisms of AD such as vascular and environmental contributions, as well as human and mouse chimeric system to incorporate human inducible pluripotent stem cell (iPSC) systems into mouse models through transplantation approaches (Fig. 1).

**Table 1:** Summary of 8 commonly used AD transgenic mouse models

**Name**	**Mutation**	**Phenotype/Pathology**	**Sex Differences**	**Behavior**	**Additional**	**Ref**
Tg2576	hAPP 695	Dense plaque (7–8 mo); major plaque deposition (11–13 mo) on parenchyma and vascular structures	Female: more plaques and congnitive impairment. Decreased REM sleep and delayed sleep onset (22 mo). Male: more aggressive. Disruped sleep EEG rhythms (22 mo).	Impairment of spatial and working memory (9–12 mo). Electrically evoked seizure (12–14 mo). Decrease of frequency of burrowing prior amyloid plaques (3 mo).	High lethality in certain genetic background. Lack of tau pathology.	[8–29]
TgCRND8	Double mutant: hAPP 695 (KM670/671 NL and V717F)	Aβ40 levels stabilized and Aβ42 increased (4–10 weeks). Amyloid deposition in the cerebral cortex (2–3 mo). Dense-cored plaques and neuritic pathology in the hippocampus, midbrain, brainstem and cerebellum (4–5 mo). Metabolic impairment, reduced NAA levels in hippocampus and cortex (2–3 mo) and dysregulation of myo-inositol levels througout mice aging.	Females: earlier learning and memory deficits (4 mo); Male: reduced REM and NREM sleeps (3–8 mo), and decreased NREM sleep (11 mo); Compensated for stereotypic behaviors (5 mo).	Disrupt in sleep cycle. Metabolic disturbance.	Aggressive model. Lack of tau pathology. Shortened life span.	[27, 30–41]
PS19	hMAPT (P301S) - mixed background	Tau seeding (1.5 mo). NFTs (6 mo). Neurodegeneration begins in hippocampus and entorhinal cortex (9 mo).	Female: higher survival rate	Impairment in memory and learning, limb weakness, hyperactivity (3 mo). Paralysis (7 mo)	No amyloid pathology. Shortened life span.	[42–45]
	hMAPT (P301S) - congenic background	NFTs (6 mo). Median lifespan of 11–15 mo.	N/A	Hyperactivity (3 mo). Altered pain perception and startle response (3 mo).	Less variability in pathologenesis compared to mixed background	[45–48]
rTg4510	hMAPT P301L crossed with a tTA allele	Pre-tangles develop (2.5 mo). Argryophilic tangle-like inclusions (4–5.5 mo). Robust tau hyperphosphorylation, neuronal loss and tangle formation (5.5 mo)	Female: more hyperphosphorylated tau, but not for tau transgene expression. Worsened deterioration of spatial learning and memory	Decline in spatial memory function (4 mo). Hyperactivity and increased anxiety (7 mo)	Pathology restricted to the cortex and hippocampus. Robust tau expression and neurodegeneration. endogenouse mouse gene disruption	[49–57]
P301L	Human 4R/2N introduced to P301L mutation	NFTs without axonal dilations in brainstem and spinal cord (6 mo). Lifespan of 8–12 mo.	N/A	Impairments in passive avoidance test (5 mo) and object recognition test (9 mo). Motor deficits (7 mo).	Of younger mice, P301L mice may have better cognitive abilities compared to wt controls.	[58–60]
APP/PS1	Double mutant: hAPP695 (KM670/671 NL) and PS1 (delEx9)	Aβ deposits, microglial and astrocytic activation (4 mo). Amyloid plaques in hippocampus and cortex (9 mo). Modest neuronal loss adjacent to amyloid plaques and synaptic dysfunction (8–10 mo). Increase in Aβ40 and Aβ42 in hippocampal regions of Nrf2 KO mice and increase in microglial activation and an accumulation of endosomes and lysosomes.	No apparent differences reported in cognitive deficits.	Memory deficits (6 mo); Deficits in spatial navigation and learning (12 mo). Nest-building and burrowing (8–14 mo).	Lack of tau pathology. LTP impairment (8–10 mo) but no PPF deficits (8–9 mo). Hippocampal neuronal circuit dysfunction.	[61–77]
5xFAD	Five mutations: Human Swedish, London, Florida APP mutations in APP and M146L and L286V in PS1 genes - Tg6799, Tg 7092, Tg 7031	Amyloid aggregates (1.5 mo). Amyloid plaques (2 mo) in hippocampus and cortex. Neuroinflammation phenotypes with atrogliosis and microgliosis (2 mo). Progressive neuonral loss (6 mo). Dystrophic neurites plateued (8–12 mo). High vs medium vs low expression	Female: more severe amyloid pathology. Male more significantly reduced PPF nad decreases in HDL levels.	Impaired spatial memory (4 mo). Motor impairments (9 mo). Reduced anxiety, increased hyperactivity (12 mo)	LTP deficits (12 mo). Lack of tau pathology. Molecular signatures are well aligned with human AD brains.	[78–86]
	5xFAD (C57BL6)	Amyloid plaques in subiculum and layer V pyramidal neurons (16 days). Intraneuronal plaques (6 weeks). Plaques in cortex, hippocampus, thalamus (2 mo) and spinal cord (3 mo). Thinner myelin sheathes (1 mo) and shorter axon calibers (2–3 mo). Loss of 40% of layer V pyramidal neurons (12 mo).	N/A	Impaired memory in cross-maze test and reduced anxiety in elevated plus maze (3–6 mo).	Lack of tau pathology. Aggressive onset of amyloid pathology.	[87–89]
	5xFAD (AD-BXD)	Varying amyloid pathology and cognitive impairment, which did not correlate.	No difference in amyloid pathology or transgene expression. Females demonstrated more motor impairments compared to males.	Varying behavior and cognitive function. Impaired function due to age and existence of transgene.	Used to model the genetic variation in humans and to identify transcriptional networks protective against AD-related cognitive decline.	[90]
3xTg-AD	Triple mutant: hPS1 (M146V), hAPP (KM670/671 NL), and MAPT P301L	Extraceullular amyloid deposits in frontal cortext (6 mo); Plaques in hippocampus (12 mo). Aggregates of conformationally-altered and hyper-phosphorylated tau in hippocampus (12–15 mo).	Female: earlier development of plaques and tangles (12 -18 mo) associated with pronounced cognitive decline, and significant age-dependent increases in microglial activation. Male mice: Markers of neuroendocrine aging appeared earlier and increased morbidity/mortality rates.	Impairment with spatial learning and memory deficits (6 mo). Age depedent cognitive decline noticed at 6, 12, and 20 mo.	Intraneuronal Aβ immunoreactivity (3–4 mo). Lack of neuronal loss. Genetic drift observed within this model.	[9, 91–96]

A summary of phenotypic characterization including neuropathological and behavioral features, sex differences in phenotype manifestation, as well as additional features is provided for 8 AD transgenic mouse models commonly used in the field including Tg2576, CRND8, PS19 (two strains), rTg5410, P301L, APP/PS1, 5xFAD (three strains), and 3xTg-AD mouse models

**Table 2:** Summary of 6 KI/KO mouse models of AD

**Name**	**Mutation**	**Phenotype/Pathology**	**Behavior**	**Additional**	**Ref**
APP KI	APP NL	N/A	Increase of anxiogenic-like behavior (15 mo).	Lack of tau pathology and no decline in spatial learning and memory.	[97–101]
	APP GF	Initial Aβ deposition (4 mo); Aβ deposition in a much larger brain area than in APP NLF or APP NLGF mice (12 mo).	N/A	N/A	
	APP NLGF	Cortical Aβ amyloidosis (2 mo) and saturated by 7 mo. Consistent subcortical amyloidosis (4 mo). Greater microgliosis and astrocytosis than NL-F mice (9 mo).	Decline in spatial learning but retained memory (8 mo). Anxiolytic-like behavior (15 mo). Hyper-reactivity to pain stimuli (15–18 mo).	N/A	
	APP SAA	Amyloid deposition detectable (4 mo). Increase of total brain density of Aβ plaques with highest burden in the cortex and hippocampus (8 mo).	Robust hyperactivity (18 mo).	Female: more pronounced hyperactivity (8 mo).	
	APP NL-F	High production of Aβ42 with the highest ratio of Aβ42/Aβ40. Initial deposition of Aβ (6 mo). Cortical amyloidosis (24 mo). Accumulation of microglia and activated astrocytes, and neuroinflammation near Aβ plaques.	Memory impairment (18 mo).	Provide a better frame for upstream factors that affect Aβ amyloidosis than other mutations.	
Tau KI	Exons 1 to 14 of mMAPT replaced with hMAPT	Normal axonal localization of tau.	N/A	Little difference compared to WT in phenotype. Often crossed with other models. MAPT P290S KI developed murine tau aggregates.	[102–104]
	MAPT KI x APP NLGF	Faster spread of pathological tau (19 mo). Tau humanization did not affect A beta or neuroinflammation.	Tau humanization did not affect memory	More tau accumulation in the presence of amyloidosis. APP NLGF x MAPT P290S dKI mice demonstrated more tau inclusions than age matched MAPT P290S KI mice. dKI mice also demonstrated tau seeding abilities.	
	5xFAD x MAPT KI	Tau humanization suggested to have a protective effect against AD. Seemed to offset LTP impairment compared to WT.	Decrease of anxiogenic-like behavior and better spatial learning compared to 5xFAD	Enrichment in lysosome, phagocytosis,and ocidative phosphorylation by GSEA compared to 5xFAD and human co-expression modules.	
APOE KI	Target replacement ApoE KI	E4FAD accumulation of Aβ42, tau hyperphosphorylation (1–4 mo), neuronal loss, deterioration of BBB, and reduced cerebral blood flow compared to E3FAD mice.	Female APOE4 KI mice have significant deficits in learning and memory. E4FAD mice developed hippocampal-associated memory deficits and had a substantial drop in nest construction scores compared to E3FAD mice.	Female: E3FAD and E4FAD have significantly higher Aβ42 and Aβ40 levels than male counterparts. Female E4FAD more deficits in learning and memory. Other deficits such as phospholipid and cholesterol dysregulation, microglial dysfunction, neuroinflammation, and taupathy-related neurodegeneration.	[105–128]
	Floxed APOE KI	APOE protein found in astrocytes but not in reactive Iba-1 positive microglia.	N/A	Typically used to cross with other transgenic models like APP/PS1 mice or PS19 mice. Overall cerebral accumulation of amyloid plaques in APOE4 KI mice crossed with APP/PS1 mice was not affected. PS19-E4 crossed mice demonstrated higher degree of neurodegeneration.	
	APOE KI: JAX	Female APOE4 KI JAX mice had lower plasma Aβ42 and a decreased Aβ42/40 ratio. However, there were no differences between APOE4 and APOE3 KI mice. Aβ40 levels did not differ regardless of APOE genotype or sex.	Locomotor activity, motor coordination, and working memory tested by open field, rotarod, and Y-maze tests, respectively were similar between APOE4 KI and control mice with an age-depedent decline (2 mo and 12 mo).	It is suggested that there are higher levels of aggregate-prone Aβ42 in the brain in female APOE4 KI mice compared to their male counterparts.	
TREM2 KO	Del exons 3 and 4	Reduced microglial numbers and size, decreased myelin repair. Prolonged microgliosis, impaired cholesterol transport.	Decreased performance on motor coordination tests (12 mo) when fed with CPZ	No apparent neurological phenotypes except for impaired immune response and altered transcriptome. TREM2 deficiency increased early-stage plaque growth, but not overall plaque deposition in an APP/PS1 dE9 mouse model with human APOE3 or APOE4.	[129–139]
	TREM2 KO x 5xFAD; TREM2 R47H x 5xFAD	High levels of amyloid in hippocampus and reduced IBA1 expression near plaques (8 mo).	N/A	Microglia are less viable than TREM2 + / + 5xFAD mice with reduced levels of CSF-1. Decreased TREM2 shedding with imparied downstream signaling in TREM2 R47H x5xFAD mice.	
	TREM2 KO x PS19	Less neurodegeneration and microgliosis compared to PS19 mice. No differences in p-tau levels and tau solubility.	N/A	Decreased inflammatory markers. Suggests that severe microglia response can contribute to neurodegeneration.	
hTREM2 KI	TREM2 CV and TREM2 R47H by Song et al.	Impaired lipid sensing and DAM responses to amyloid. Impaired soluble TREM2 cell-surface interactions with decreased TREM2 shedding noted on neurons.	N/A	Mice developed less brain atrophy and synaptic loss with diminished microglial reactivity and phagocytosis when compared to PS19-TREM2(CV) mice.	[132, 133, 140–148]
	TREM2 R47H and APPPS21-TREM2 + /R47H by Cheng-Hathaway et al.	Attenuated microglial response to amyloid with reduced amounts of dense-core plaques. TREM2 R47H mice with cuprizone-induced neuro-inflammation demonstrated age-depedent impairments in microglial interaction with plaques (4 mo), and LTP and synaptic loss (12 mo).	N/A	Attenuated microglial response to amyloid with increased neurite dystrophy.	
	LOAD1, LOAD2 and others by JAX	No amyloid plaques or other AD hallmark changes observed in LOAD1 mouse models even at 24 months of age. After high fat diet (HFD) treatment, LOAD2 mice demonstrated neuronal loss and elevated brain Aβ42 (16 mo).	No cognitive deficits observed in LOAD1 mouse models even at 24 months of age. LOAD2 mice on HFD exhibited behavioral deficits.	Reduction in brain TREM2 protein levels and changes in circulating cytokine levels. Regional changes in glycolysis and vascular perfusion. Female LOAD1 mice showed increased risks of mortality and glycolysis was significantly altered (4 mo-12 mo).	
hTREM2 Tg	BAC TREM2 Tg	Reduced mamyloid plaques with associated gene signature changes including dampened damage-associated microglial gene expression and up-regulated neuronal gene expression.	Cognitive performance improved in BAC hTREM2 Tg x 5xFAD mice compared to 5xFAD mice with increased phagocytic microglia and reduced neurite dystrophy seen.	N/A	[149–151]

A summary of phenotypic characterization including neuropathological and behavioral features, as well as additional phenotypes is provided for 6 AD KI/KO mouse models recently developed in the field including APP KI (5 different APP KI), Tau KI (with additional crossing models), APOE KI (three strains), TREM2 KO mouse models (three strains), hTREM2 KI mouse models (three strains with additional crossing models), and BAC hTREM2 Tg mouse models

### Transgenic mouse models of Alzheimer’s disease

#### Amyloid Tg mice

##### Tg2576

The Tg2576 model expresses the 695-amino acid isoform of human amyloid precursor protein (APP) with the Swedish mutation inserted into the hamster prion protein (PrP) coplasmid vector resulting in a fivefold increase in Aβ_40_ and a 14-fold increase in Aβ_42_/Aβ_40_. Around 6–7 months of age, Tg2576 mice were found to accumulate Aβ_40_ and Aβ_42_ species that were Sodium Dodecyl Sulfate (SDS)-resistant. At 7–8 months of age, amyloid plaques became dense and visible with a wide spread of plaque build-up and deposit on mouse brain parenchyma as well as vascular structures by 11–13 months of age [10]. It was also found that female Tg2756 were more susceptible to developing plaques than their male counterparts [9].

Unlike most other APP models, cognitive decline in Tg2576 mice manifested months prior to pathology whereas cognitive decline occurred in close proximity in other models [8]. Cognitive impairment in Tg2576 mice can be manifested as impaired spatial and working memory measured by behavioral tasks such as the Y-maze, the Morris water maze and the contextual fearing conditioning tests [10–12]. Some reported cognitive deficits as early as 6 months of age but most studies reported cognitive dysfunction starting 9–10 months of age and progressively noticeable after 12-month of age [10–12]. One study reported a decreased frequency of burrowing in Tg 2576 mice that can be seen as early as 3 months of age preceding the formation of amyloid plaques [13]. Sex-specific differences in cognitive impairment were noted with a rapid progression in females [14], as well as a greater degree of cognitive impairment observed in female mice [9] with the Tg2576 mouse model.

Besides cognitive deficits, other behavior disturbance was reported in the Tg2576 mouse model such as non-rapid eye movement (NREM) sleep disorder and an increased susceptibility to seizure. At 22 months of age, male Tg2576 mice had disrupted sleep EEG rhythms and female Tg2576 mice had decreased REM sleep and delayed sleep onset [15]. It was speculated that cholinergic dysfunction may contribute to sleep and circadian rhythm abnormalities [16]. Moreover, when compared to wildtype (WT) littermates, Tg2576 mice at age of 12–14 months old were more susceptible to electrically evoked seizures [17], and an increased sensitivity to kindling epileptogenesis [18]. One study reported a high susceptibility to audiogenic-induced seizures in Tg2576 mice that were reduced by passive immunization of an anti-Aβ antibody therapy [19]. Overall, this increased susceptibility to seizures may contribute to a higher mortality rate with Tg2576 mice.

While synaptic loss was absent in Tg2576 mice, changes in synaptic plasticity were reported with impaired LTP measured in the dentate gyrus and CA1 region of hippocampus [20]. In the hippocampus and cortex of aged Tg2576 mice, reduced cholinergic receptor binding and decreased choline uptake were observed supporting cholinergic dysfunction [21, 22]. There were dystrophic cholinergic fibers associated with amyloid plaques [23, 24]. Moreover, cortical neurons derived from Tg2576 mice were found with an impaired retrograde trafficking of BDNF leading to cholinergic degeneration [25, 26].

Besides the absence of a widespread cell loss, Tg2576 mice did not show any build-up of neurofibrillary tangles either [27]. In general, the Tg2576 mouse model is considered as a modest neurodegenerative model possibly due to the promoter used in over-expressing APP [28]. This model was noted with high lethality in certain genetic backgrounds and males tended to be aggressive that required single housing [9]. However, this model is reasonably suited for understanding the pathogenic processes of amyloid in AD [29].

##### TgCRND8

TgCRND8 mice encode a double mutant human APP 695 (the Swedish mutation KM670/671 NL and the Indiana mutation V717F) under the hamster prion protein promoter (PrP) with 5-fold of human APP over-expression [27]. Aβ_40_ levels were stabilized between 4–10 weeks of age whereas Aβ_42_ increased slowly between 4–8 weeks with a potent increase at 10 weeks of age [27]. At 2–3 months of age, amyloid deposits were seen in the cerebral cortex. Two months later, dense-core plaques and neuritic pathology began to show in the brain regions of hippocampus, midbrain, brainstem and cerebellum [30]. Interestingly, while TgCRND8 males and females exhibited equivalent Aβ pathologies at 2–8 months of age, females displayed learning and memory deficits much earlier than males [31].

It was reported that TgCRND8 mice manifested with learning impairment detected by the Morris water maze studies as early as 11-week of age that was offset by immunization against Aβ_42_ [32]. Sex-dimorphic behavioral deficits were described as well. For example, female TgCRND8 mice at 4 months of age had learning and memory deficits detected by the novel object recognition tests [33]. In addition, others described that TgCRND8 males at 5 months of age compensated for Aβ-associated stereotypic behaviors such as hyperactive tight cycling by alternating navigational search strategies and increasingly productive spatial search strategies while females failed to do so [31]. Moreover, sleep-wake cycle dysfunction associated with amyloidosis was reported in male TgCRND8 mice with reduced REM and NREM sleeps at 3–8 months of age, as well as decreased NREM sleep only at 11 months of age [34].

Besides amyloid pathology and behavior impairment, metabolic disturbance was reported in TgCRND8 mice using ^1^H NMR spectroscopy methods [35]. It was found that levels of N-acetylaspartate (NAA) were reduced in brain regions such as hippocampus and cortex even in young Tg mice (2–3 months old) compared to wt controls prior to any detectable pathological changes, and levels of *myo*-inositol were decreased in cortical brain regions of young Tg mice but increased in older (12–13 months old) Tg mice [35]. NAA is a metabolite considered to reflect neuronal mitochondrial function [36, 37]. Prior studies implicated a correlation between reduced NAA levels with brain pathology and disease progression in AD patients [38]. It has been found that a decrease in NAA and an increase in *myo*-inositol both occurred during neuronal cell loss or dysfunction and associated gliosis [39, 40]. These observed changes in NAA and *myo*-inositol levels suggested underlying neuronal dysfunction or cell loss with associated gliosis [35].

In general, the TgCRND8 mouse model is considered an aggressive model of brain amyloid deposit without tangle pathology. It should be noted that prior report described differences in survival rates and amyloid burden based on different genetic backgrounds in this mouse model with a noticeably shortened life span and a decreased survival rate of TgCRND8 mice with a B6 background but an improved survival rate with less amyloid burden in mice with an A/J inbred background [41]. These findings implicate a layer of complexity with genetic heterogeneity of AD mouse models which could potentially contribute to reported variabilities in AD-related phenotypes and findings in some AD mouse models.

#### Tau Tg mice

##### PS19

This mouse model carries human MAPT (1N4R) tau P301S mutation, under the mouse Prp promoter. This model recapitulates many of the major phenotypes of AD, such as neurofibrillary tangles, synaptic dysfunction, cognitive impairment, and neuronal loss. In this model, human tau expression levels were 5 times that of endogenous mouse tau [42]. There are two primary strains of PS19 mice, namely the mixed background B5;C3 mice and the mixed mice backcrossed with C57BL/6J mice to create a congenic background.

The mixed PS19 mouse model demonstrated pathological tau seeding as early as 1.5 months of age. Tau pathology was developed along neural networks, suggesting tau spread in a prion-like mechanism through neural connections [43]. The initial neuropathological manifestation also included gliosis and impaired synaptic function, followed by synaptic loss. Axonal dysfunction was seen with compromised ER transport as early as 3 months in PS19 mice. At 4 months of age, PS19 mice were positive for neuro-inflammatory markers. These changes occurred prior to the development of tau pathology [42].

The transgenic mice developed neurofibrillary tangles at 6 months of age, and hippocampal and entorhinal cortical atrophy at 9–12 months of age. Specifically, at 12 months of age, there was a 20% reduction in cerebral cortical volume, and a 45% reduction in hippocampus volume. The median life expectancy of PS19 mice was 9 months with 80% of mice dying by 12 months [42]. In a separate study, it was found that female PS19 mice had a significantly higher survival rate compared to male PS19 mice, 90% *versus* 32% of survival rate at 12 months of age [44]. Interestingly, when 2 months old PS19 mice were immuno-suppressed with FK506 treatment, the survival rate of mice at 12 months of age was increased from 20 to 60% with an associated decrease in neuro-inflammatory markers, neuronal loss, and insoluble hyper-phosphorylated tau. These findings suggest that irregular microglial activation in PS19 mice may exacerbate the effects of tau pathologies and thereby contribute to disease progression [42]. PS19 mice exhibited impairment in memory and learning abilities as early as 3 months of age, before much of the pathology appeared. Mice soon developed limb weakness and later paralysis by 7 months of age [42]. In the Morris water maze test, PS19 mice spent a much longer time finding the invisible platform [45].

The congenic line demonstrates less variability in disease progression, developing neurofibrillary tangles at 6 months and neuronal loss at 9 months of age [46]. They have a median lifespan of 11 to 15 months. Congenic transgenic mice showed a significantly increased tendency to go into and spend time on open arms, suggesting hyper-activities in these mice. It was also reported that PS19 mice showed significantly lower anti-nociceptive responses, with lower thresholds in the hot plate test and lower startle amplitudes in the pre-pulse inhibition test. However, PS19 mice at 3 months of age demonstrated no differences in the tendency to fall when compared to wt counterparts as measured in the rotarod test, suggesting a relatively functional motor tract of these mice at early ages [45].

While the PS19 mouse model simulates many of the AD phenotypes, it does not manifest amyloid pathology. With the high mortality rate in early ages, it is challenging to study tau pathology in this mouse model in later time points. Despite the drawbacks, this model has been used to test novel drug candidates targeting tau pathology, such as microtubule stabilizers like Epothilone D, or lead compounds that reduce tau hyperphosphorylation [47, 48].

##### rTG4510

Similar to the PS19 model, the rTG4510 model over-expressed a human frontotemporal dementia (FTD)-associated MAPT tau mutation. Instead of the P301S tau mutation in PS19, rTG4510 over-expressed the P301L tau mutation with an accelerated formation of a unique 64 kDa hyper-phosphorylated 4R0N isoform of tau. The mouse model was generated by crossing a responder line containing human MAPT P301L cDNA, and a separate line with the tetracycline-controlled trans-activator (tTA) allele under control of the forebrain-specific CaMKIIα promoter. Therefore, human mutant tau transgene expression was largely limited to the hippocampus and neocortex, with associated deficits in hippocampal related activities. It was referred to as the “regulatable” TG4510 as transgene expression can be regulated through doxycycline chow feeding, which provided a temporal control of mutant tau transgene expression [49, 50].

rTg4510 mice expressed 13 times higher of transgenic tau levels compared to endogenous mouse tau levels, forming pre-tangles at 2.5 months of age, and argyrophilic tangle-like inclusions by 4–5.5 months of age in the cortex and the hippocampus [49, 50]. Interestingly, female mice manifested earlier but more severe tau pathology compared to male mice, with significantly increased levels of hyperphosphorylated tau at 5.5 months of age, despite no sex differences in levels of tau transgene expression [51].

A rapid neuronal loss was seen along with tangle formation by 5.5 months of age, and ~60% of decrease in hippocampal CA1 neurons and cortical cell loss occurred at 8.5 months of age [49, 50, 52]. With the progression of tau pathology, axonal degeneration as well as demyelination and impaired white matter integrity were noted by electron microscopy studies [53, 54]. Notably, when suppressing transgenic tau expression through doxycycline chow feeding for 6–8 weeks, CA1 neuronal loss was stabilized, and brain volume loss was prevented after 5.5 to 9 months of feeding. Halting transgenic tau expression at 2.5 months of age stopped the progression of tangle formation and neuronal loss. However, if tau suppression was initiated at 4 months or older, tangle formation proceeded but neuronal loss was prevented suggesting that NFTs may not directly cause neuronal death [50]. It was also found that PS19 mice at 5 months of age had a reduced synchronization of excitatory neurons, which impeded downstream neuronal depolarization and firing. Specifically, membrane potential oscillations during the slow-wave sleep were slowed with altered firing patterns. While few neurons contained NFTs at this age, young mice still experienced spatial memory deficits, indicating that soluble tau may be a primary source of synapse damage, supporting the conclusions from prior studies [55].

Behavioral studies indicated an age-dependent decline in spatial memory function as detected by the Morris water maze tests, severely compromised in rTg4510 mice by 4 months of age. At 7 to 9.5 months of age, Tg mice demonstrated random swimming, with only 25% of time spent in the target quadrant [49, 50]. Additionally, Tg mice displayed a heightened hyper-activity as detected in the open field and elevated plus maze tests. On the other hand, mice showed an increased tendency to freeze in the open arms and light chambers in the elevated plus maze test, suggesting an increased anxiety trait [56]. Sex differences in behavioral deficits have been reported as well. For example, female Tg mice exhibited worsened deterioration of spatial learning and memory, showing markedly worse learning acquisition, with longer search paths in the Morris Water maze tests. They also showed much lower memory scores than their male counterparts, despite comparable memory scores between male and female non-Tg mice [51].

It should be noted that recent studies suggested that factors other than hTau expression may contribute to phenotypes observed in rTg4510 mice. The CaMKIIa-tTa and MAPT transgenes were found to disrupt endogenous mouse gene expression such as fibroblast growth factor 14 (Fgf14), which may cause several neurodegenerative phenotypes [57]. Additional layer of complexities comes from the regulatable tau transgene expression in this mouse model. Overall comparing to the PS19 mouse model, the rTg4510 mouse model manifested an earlier onset and increased severity of tau pathology. Moreover, tau pathology in this mouse model is more concentrated near the cortex and hippocampus, as opposed to the brainstem and spinal cord in the PS19 mouse model.

##### P301L Van Leuven model

The Van Leuven model was generated by introducing the human 4R/2N isoform of tau with the P301L mutation under the thy-1 murine gene promoter. The main purpose of the model was to assess the effects of the P301L mutation in the context of human tau. These mice exhibit NFTs without axonal dilations by 6 months of age primarily within the brainstem and spinal cord [58]. These mice generally have a lifespan between 8 and 12 months of age [59] and demonstrate both cognitive and motor deficits [58, 60]. Significant deficits in the passive avoidance test begin at 5 months, and deficits in the novel object recognition test begin at 9 months. At 7 months, mice develop motor deficiencies as well as increased clasping of limbs [58]. However, some evidence suggests that of younger mice, before aberrant conformational changes in the brain and cognitive deficiencies, P301L mice demonstrate better cognitive abilities compared to wt controls. For example, P301L mice exhibit enhanced object recognition memory and LTP, which is hypothesized to be due to enhanced aging in dendritic spine in P301L mice [60].

#### Double/triple Tg mice

##### APP/PS1 - Borchelt mice

This model, commonly referred to as “Borchelt mice”, was created by co-injecting Swedish APP695 mutations (KM670/671 NL), as well as PS1 sequence lacking exon 9 (ΔE9) [61]. Both transgenes were inserted into the mouse PrP promoter in a single locus causing an 1 bp duplication that did not affect any known genes [62].

It was reported that Aβ deposits can be seen as early as 4 months of age with an increase in microglial and astrocytic activation. By 9 months of age, an increasing abundance of amyloid plaques can be found in the hippocampus and cortex [61]. Between 8 and 10 months of age, modest neuronal loss was observed adjacent to amyloid plaques [63]. However, tangles were not seen in these animals. Regulation of autophagy pathway was also investigated using the APP/PS1 mouse model with NF-E2 related factor 2 (Nrf2) knock out to inhibit autophagy with a significant increase in mTOR activation [64]. Nrf2 is an emerging regulator of cellular resistance to oxidants which helps control the physiological and pathophysiological outcomes of oxidant exposure [65]. It was found that in Nrf2^−/−^ APP/PS1 mice, there was an increase in Aβ_40_ and Aβ_42_ in hippocampal regions compared to Nrf2^+/+^ APP/PS1 mice. There was also a significant increase in total human Aβ_42_ levels in knockout mouse hippocampal regions. In addition, there was an increase in microglial activation, as well as an accumulation of multivesicular bodies, endosomes and lysosomes [64]. It has been shown that vesicle accumulation leading to the degradation of neurons [66–68]. Moreover, by combining MRI-based morphometry and MRS-based analysis, studies demonstrated that APP/PS1 mice presented with an increase of amyloid plaques, neuronal loss, and an impairment of NAA/Cr ratio, however, brain atrophy was absent [69]. Proteomics studies of 12 months old APP/PS1 mouse brains showed up-regulation of protein expression involved with APP processing and Aβ formation, such as Retention in Endoplasmic Reticulum 1 (RER1), as well as highly up-regulated protein expression associated with lysosome function, such as Hexosaminidase Subunit Beta (HEXB), Lysosomal Associated Membrane Protein-2 (LAMP2), and Phospholipase D Family Member 3 (PLD3) [70].

Compared to other mouse models such as rTg4510 mice, APP/PS1 mice exhibited a slower decline in synaptic function. In fact, synaptic dysfunction was not seen until later age like 8–10 months of age in the APP/PS1 mice [71]. Short-term plasticity plays a critical role in neuronal information processing. It was found that APP/PS1 mice did not show any deficit in paired-pulse facilitation (PPF) at least up to 8–9 months of age whereas rTg4510 mice exhibited reduced PPF at 6–7 months of age suggesting abnormalities in presynaptic release machinery. It is speculated that rTg4510 mice had reduced or eliminated synaptic facilitation while APP/PS1 mice did not [71]. Long-term potentiation (LTP), a cellular/molecular correlate of memory was impaired in both mouse models with APP/PS1 mice manifested at 8–10 months of age and rTg4510 mice at 6–7 months of age. These LTP deficits, however, were attributed differently across these two models. In rTg4510 mice, LTP was attributed to altered postsynaptic signaling pathways while in APP/PS1 mice, it was attributed to induction deficits [71].

Memory deficits such as impaired contextual fear conditioning behaviors were observed in the APP/PS1 mice as early as 6 months of age [72], with deficits in spatial navigation and learning as measured by the Morris water maze tests by 12 months of age [63, 73, 74]. Spontaneous behaviors such as nest-building and burrowing were also affected at the ages of 8–14 months of age [75]. However, sex differences in behavior deficits were not noted or described in this mouse model. For example, it was found that there were no sex-dependent differences in cognitive deficits within APP/PS1 mice as measured by the Banes maze tasks [76].

Interestingly, it was noted in APP/PS1 mice that Aβ had different effects on hippocampal neuronal circuit function depending on the brain states associated with memory encoding and consolidation. Aβ increased neuronal activities during the wakefulness and the NREM sleep but suppressed activities during the quiet wakefulness and the REM sleep. It was also suggested that Aβ deposition in the hippocampus may lead to hippocampal neuronal hyperactivity in the wakefulness and impair theta-gamma phase amplitude coupling (PAC), a proposed biomarker of AD. Reducing theta-gamma PAC during the REM sleep may contribute to hippocampal dependent memory impairment in AD [77].

Due to the fact that these mice developed such apparent Aβ pathology and memory deficits as early as 6 months of age, the APP/PS1 model is valuable in studying the development of new therapeutic approaches targeted against amyloid as well as AD-related microglial dysfunction and vesicle trafficking impairment.

##### 5xFAD

The 5xFAD model is a very commonly used AD mouse model that accounts for 10% of all AD studies employed mouse models. It combined human Swedish, London, and Florida APP mutations with PS1 M146L and L286V mutations, leading to over-expression of a total of five AD-linked mutations under the mouse Thy1 promoter. Compared to APP mutations only mouse models, this mouse model produced robust and accelerated amyloid pathology with no tau pathology [78]. When the 5xFAD mouse model was first introduced, high (Tg6799), medium (Tg7092), and low (Tg7031) levels of APP over-expression lines were generated, with the “high” line being most used. All lines showed a general trend of generating Aβ_42_ almost exclusively, with Aβ_40_ lagging a few months behind. Comparatively, the Tg6799 line displayed the highest amounts of Aβ_42_ accumulation, while the Tg7031 line had the lowest with a later age of onset of AD pathologies [78].

In general, 5xFAD model mice developed many AD-related phenotypes much earlier than most other amyloid based mouse models. Tg mice accumulated intraneuronal Aβ_42_ aggregates in the cerebrum starting at 1.5 months of age. Extracellular Aβ plaques can be observed at around 2 months and increase rapidly with age, first in the subiculum of the hippocampus and layer V of the cortex. Plaques were seen throughout the hippocampus and cortex by 6 months of age. Additionally, amyloid pathology was more severe in females than in males, possibly because of an estrogen response element in the Thy-1 promoter [78–80]. Female mice also had a more intense increase in hippocampus plaque burden in response to behavioral stress [81]. The initial morphology of amyloid plaques had a compact circular form then became more irregular and diffuse around the subiculum, CA1, and cortex around 12–18 months of age, mirroring what had been observed in the human brains [82].

The 5xFAD mice exhibited synaptic dysfunction and impaired long-term potentiation at 4 months, as well as astrogliosis and microgliosis as early as 2 months of age, suggesting a much earlier neuro-inflammation phenotype compared to other AD mouse models [78, 82]. Mice also experienced an increase in proinflammatory cytokines such as IL-1B, TNF-a, and KC/GRO levels [83]. Progressive neuronal loss began at 6 months of age in the areas with most pronounced amyloidosis, and then developed in multiple brain regions. Dystrophic neurites by immunostaining of LAMP-1 were increased with age but plateaued at 8–12 months of age [82]. At 12 months of age, the potentials of theta burst LTP and the fEPSP responses were significantly reduced in both male and female 5xFAD mice, whereas the frequency of paired pulse facilitation were significantly reduced only in male 5xFAD mice [82]. Male 5xFAD mice also exhibited an age-related decrease in HDL levels compared to WT counterparts, which may be associated with an increased risk for neurodegeneration [83].

Behavior impairments were seen in 5xFAD mice with impaired spatial working memory measured by the Y-maze test by 4–5 months of age [78, 81], as well as impaired spatial memory at 4–5 months of age detected by the Morris water maze tests [84] and contextual-fear-conditioning tests [85]. Additionally, Tg mice exhibited motor impairments that were apparent by 9 months of age, performing poorly on the Rotarod test and balance beam [86]. Moreover, 5xFAD mice at 12 months of age spent more time in the open arms of the elevated plus maze, and in the middle of the open field test suggesting a reduced anxiety but an increased hyperactivity [82]. These mice failed to gain weights at 8 months of age, an observation most significantly affecting female mice [82].

Transcriptomic analysis of 18 months old 5xFAD mouse brains revealed a significantly increased number of up-regulated differentially expressed genes (DEGs), mostly involved in neuro-inflammation, with substantial overlap between hippocampus and cortex up-regulated genes. There were fewer down-regulated DEGs, mostly associated with synaptic transmission and signaling [82]. A more detailed analysis of transcriptomics dataset of various AD mouse models including this study will be discussed in the later section.

**5xFAD (C57BL6)**

This mouse model, bred on a C57BL6 background, developed amyloid plaques most aggressively in the subiculum and in layer V pyramidal neurons as early as 16 days of age and intraneuronal plaques at 6 weeks old [87]. Plaques appeared in the cortex, hippocampus, and thalamus by 2 months of age [87], and in the spinal cord by 3 months of age [88]. By 1-month of age, mice demonstrated thinner myelin sheathes, and by 2 to 3 months old, they demonstrated shorter axon calibers [89]. By 12 months of age, mice lost 40% of layer V pyramidal neurons [88]. Mice showed impaired spatial working memory in the cross-maze test, as well as reduced anxiety in the elevated plus maze at 3 to 6 months of age [88].

**5xFAD (AD-BXD)**

This mouse model was created by breeding female 5xFAD mice with BXD mice, transgenic mice created to mimic the genetic diversity of humans. They had varying levels and ages of onset of amyloid pathology and cognitive impairment, the two of which did not correlate. On the other hand, cognitive impairment was correlated with a genetic risk score calculated from 21 LOAD-associated genes [90].

Together, these finding support the inter-animal variabilities with different genetic backgrounds as well as different expression levels of transgenes in different 5xFAD mouse models. These popular models are known for the aggressive plaque and neuro-inflammatory pathology, allowing amyloid and other AD-related phenotypes to be studied more in-depth. This, along with extensive behavioral impairments, made 5xFAD a favorable model to test therapeutic interventions at early stages of the disease. Moreover, recent Omics studies generated from the mouse model were well-aligned with several molecular signatures of human AD brains, supporting the relevance of this model in studying human disease processes (discussed in the later section). However, this model notably lacks the presence of NFTs, along with a rapid progression of amyloid pathology, making it less suitable to study the nature disease trajectory with focuses on later stages of AD.

##### 3xTg

The triple-transgenic model carries human AD mutations including the presenilin 1 (PS1) mutation M146V, the Swedish APP mutations KM670/671 NL, and MAPT P301L tau mutation. This is the first transgenic model to develop both plaque and tau pathology in AD-relevant brain regions. Rather than crossing three independent mouse lines to achieve this triple-transgenic model, transgenes were introduced into mouse germ lines [91].

As opposed to double transgenic mouse models that carry the human APP and/or PS transgenes, the 3xTg mice develop extracellular plaque as well as intraneuronal Aβ immunoreactivity. Aβ deposition can appear in some brain regions as early as 3–4 months of age and extracellular amyloid deposits can be seen in the frontal cortex by 6 months of age. By 12 months of age, all 3xTg mice had accumulated plaques across the hippocampal regions [92]. Tauopathy occurred later - around 12 to 15 months of age – when aggregates of conformationally-altered and hyper-phosphorylated tau were detected in the hippocampus [91, 93]. On the other hand, tau pS422 immunoreactivity was detected in the caudal and medial hippocampus regions by 6 months of age. By 12–20 months of age, extensive pS422 immunoreactivity was seen in neurons across the brain regions [92]. The temporal sequence of tauopathy development being much later than amyloid pathology was supportive of the amyloid cascade hypothesis. It was also found that 3xTg female mice were more susceptible to an earlier development of plaque and NFT-like pathologies than their male counterparts [9]. It was found that there were increased levels of Aβ in female 3xTg mice at 12 months of age [94] and 18 months of age [95].

Cognitive impairment in 3xTg mice was observed at 6 months of age with spatial learning and memory deficits detected by the Morris water maze tests [92]. An age-dependent cognitive decline was reported with Tg mice performing significantly worse when compared to the non-Tg mice at 6, 12, and 20 months of age [92]. Interestingly, a study reported a correlation between cognitive impairment and the accumulation of intraneuronal Aβ in the hippocampus and amygdala when plaques and NFT-like formation were not yet apparent [93]. It was also suggested that an earlier-onset AD pathologies in female 3xTg mice may contribute to a more pronounced degree of cognitive decline and impairment in females [9]. On the other hand, there were male-specific deficits noted in 3xTg mice. It was found that markers of neuroendocrine aging appeared earlier in male than female 3xTg mice [9]. Male 3xTg mice also presented with a more vulnerable neuro-immunoendocrine network which could result in a higher susceptibility to deleterious effects of aging and accountable for the increased morbidity and mortality rates observed in male 3xTg mice compared to female counterparts [96].

One major limitation of 3xTg mice was lack of neuronal loss despite the buildup of Aβ deposits and tau pathology. However, at 6 months of age, there was a significant age-dependent increase in microglial activation in female 3xTg mice compared to non-Tg mice in hippocampal regions, implicating an advantage of studying a role of neuro-inflammation using this mouse model [92]. Another major limitation noted was a significant variability in pathology between not only sexes but between colonies. Genetic drift was observed within this model which may contribute additionally to the phenotypic heterogeneities noted in the 3xTg mice [9].

### Knock-in (KI) and knock-out (KO) mouse models of AD

#### Genetic risk factors of AD mouse models

##### APP KI

This model carries an insertion incorporating wild-type mouse APP exon 16 and exon 17. An additional copy of exon 16 carrying the Swedish mutation and a modified exon 17 with the London and Dutch mutations were crossed with a Flippase (FLP) strain. The murine Aβ sequence was humanized and then inserted with Swedish and Beyreuther/Iberian mutations [97]. Essentially, levels of Aβ_40_ and Aβ_42_ were increased with a higher ratio of Aβ_42_ without the over-expression of APP. Multiple lines of this APP KI model were created: mice inserted with Swedish mutation (APP^NL^); mice inserted with the Swedish and Beyreuther/Iberian mutations (APP^NL−F^); mice with the Swedish, Beyreuther/Iberian, and Arctic mutations (APP^NLGF^); mice with the Beyreuther/Iberian and Arctic mutations (APP^GF^); and mice with the Swedish, Arctic, and Austrian mutations (APP^SAA^).

In APP^NLGF^ mice, cortical Aβ amyloidosis began as early as 2 months and became saturated by 7 months of age [98]. It was found that homozygous APP^SAA^ mice had amyloid deposition detectable from 4 months of age and the total brain density of Aβ plaques increased from 4 months of age to 8 months of age. At 8 months of age, Aβ plaques were detected in multiple brain regions with highest burden in cortical and hippocampal regions [99]. Initial Aβ deposition was observed as early as 4 months of age in APP^GF^ mice and at 12 months of age, Aβ deposition in APP^GF^ mice was detected to be in a much larger brain area than that in APP^NL−F^ mice but in a lesser area than that in APP^NLGF^ mice [100]. It was also found that p-tau 217, p-tau 231, and a fraction of p-tau 181 were detected around Aβ plaques only in APP^NLGF^ mice but not in APP^NL^ or wildtype mice, suggesting that these tau pathologies may be induced by Aβ plaque burden [101].

Despite an early and aggressive Aβ amyloidosis in APP^NLGF^, neuroinflammatory responses were not intense at 6–9 months of age, but greater reactive gliosis was observed in cortical and hippocampal regions by 15–18 months of age [98]. On the other hand, amyloidosis in APP^GF^ mice were accompanied with neuroinflammation, as shown by reactive astrocytes and activated microglia at 22 months of age [100].

The APP^NL^ mice exhibited anxiogenic-like behaviors from 15 months of age, while APP^NLGF^ exhibited anxiolytic-like behaviors. In the fear conditioning tests, both APP^NLGF^ and APP^NL^ mice exhibited intact learning and memory up to 15 to 18 months of age, but APP^NLGF^ exhibited hyper-reactivity to pain stimuli [98]. It was also found that in the Barnes maze task, APP^NLGF^ mice exhibited a decline in spatial learning at 8 months of age but retained memory function [98]. Heterozygous APP^SAA^ mice displayed robust hyper-activity at 18 months of age and females showed a more pronounced hyperactive phenotypes as early as 8 months of age [99].

In summary, with various lines of APP KI mouse models, one of the advantages is the versatility through the combinations of various human APP mutations that can be used. On the other hand, these models still lacked development of tau pathology with relatively subtle cognitive deficits seen.

##### Tau KI

Since tau pathology in human AD is not caused by mutations in MAPT, the MAPT KI model was initially created to investigate the amyloid cascade hypothesis by observing the effects of an APP knock-in in a humanized tau environment. To accomplish this, exons 1 to 14 of murine MAPT were replaced with human MAPT sequence through a homologous recombination approach [102]. This MAPT KI mouse model differs from the MAPT mutation mouse models in that instead of artificially introducing mutant tau, it allows studying development of AD pathology and the impact of environmental factors on these pathology through recapitulation of human wildtype tau conditions. MAPT KI mice expressed all six human MAPT transcripts in replacement of mouse tau, while showing normal axonal localization of tau [102].

However, a MAPT KI x APP^NLGF^ KI model demonstrated cognitive characteristics similar to those of APP^NLGF^ KI mice, suggesting that the MAPT KI mice induced no artificial phenotypes, and that humanized tau acted similarly as murine tau. Despite the presence of humanized tau, AD phenotypes including amyloid pathology, neuroinflammation, neurodegeneration and memory deficits were all unaffected compared to APP^NLGF^ KI mice [102]. Similarly, NFTs and filamentous tau did not develop in dKI mice or APP^NLGF^ KI mice further strengthening the notion of challenges in observing tau pathology in mouse models without introducing MAPT mutation. Moreover, amyloidosis exacerbated hyper-phosphorylation of both human and murine tau at a similar rate, suggesting that human and murine tau didn’t differ in amyloidosis-induced phosphorylation. The expression ratio between 4 and 3R tau was similar between dKI and MAPT KI mice, even when the mice aged, suggesting that amyloidosis didn’t affect the alternative splicing of MAPT gene [102].

On the other hand, dKI mice demonstrated a faster spread of pathological tau compared to APP^NLGF^ knock-in mice. Additionally, more AT8^+^ tau aggregates in dystrophic neurites were seen in close proximity to amyloid plaques, but neither NFTs nor neurodegeneration were noted [102]. In a separate study, a mouse model of MAPT^P290S^ KI, the murine equivalent of human P301S mutation, was generated, and then crossed with the APP^NLGF^ line. The MAPT^P290S^ KI mice showed AT100^+^ tau inclusions, suggesting that murine tau aggregates can be formed in vivo despite isoform and amino acid differences from human tau [103]. Sarkosyl-insoluble tau was detectable in 18 months old APP^NLGF^ x MAPT^P290S^ dKI mice, with significantly more tau inclusions in these dKI mice compared to MAPT^P290S^ KI mice at similar ages, supporting that amyloid pathology facilitated the progression of tau aggregation. In addition, tau pathology was generally more noticeable near regions with amyloidosis [103].

In dKI mice, dystrophic neurites surrounding amyloid plaques were positive for AT8, AT100, and Gallyas-Braak, indicating the presence of filamentous and hyper-phosphorylated tau. At 22–24 months of age, the dKI mice, compared to MAPT^P290S^ KI mice, reached a 33-fold increase for AT100 and a 75-fold increase in AT8 filamentous tau [103]. In contrast, APP^NLGF^ mice were only immunoreactive for AT8, but not AT100 and Gallyas-Braak Silver, suggesting the presence of hyper-phosphorylated but not filamentous tau. Tau seeding abilities were examined in aged MAPT^P290S^ KI mice and the double KI, with the dKI at 18 months exhibiting 43 times higher seeding abilities, while in MAPT^P290S^ KI mice only 3 times higher seeding abilities when compared to wt counterparts [103]. The majority of tau propagation occurred in the presence of amyloid plaques, implicating an amplifying effect of amyloid for tau pathology. In the dKI mouse model, neuronal loss was significantly more than age matched MAPT^P290S^ mice from 18 months onward, which corresponded with a significant increase in tau inclusions [103]. Notably, amyloid plaques were seen intracellularly, followed by extracellular plaque buildup that were surrounded by Gallyas-Braak positive neuritic processes, astrocytic processes, and microglial cells [103].

Recent studies indicated that tau humanization may have a protective effect against certain AD related processes. A 5xFAD crossing with MAPT KI mouse model revealed MC1^+^ tau pathology, suggesting that a conformational tau pathology more closely reminiscent of human AD phenotypes. However, in the elevated plus maze tests, this mouse model spent less time in open arms than 5xFAD mice, indicating that human tau may partially rescue anxiety-like behaviors. Additionally, both MAPT KI mice with 5xFAD background and MAPT KI mice without 5xFAD background demonstrated better spatial learning abilities compared to 5xFAD mice in the Morris water maze, adding a layer of complexity to the effects of humanized tau on disease progression in 5xFAD background [104]. The MAPT KI mice with 5xFAD background also seemed to offset LTP impairments, with no statistically significant differences between these mice and wt controls [104]. Moreover, MAPT KI mice with 5xFAD background showed a negative correlation in AD gene expression when compared to 5xFAD and human co-expression network modules. Gene set enrichment analysis (GSEA) revealed a significant enrichment in processes related to lysosomal function, oxidative phosphorylation, and phagocytosis in MAPT KI mice with 5xFAD background when compared to 5xFAD mice [104].

In summary, these recent tau KI mouse models are useful for studying the effects of amyloid pathology on AD-related tauopathy without over-expressing human tau like tau transgenic models. However, the failure of developing NFTs in APP^NLGF^ x MAPT dKI mice highlights the challenges of naturally modeling tauopathy that aligns with clinical AD development chronologically, when tauopathy develops decades after amyloidosis. While NFTs were observed in APP^NLGF^ x MAPT^P290S^ dKI mice, the relevance to human AD is limited with the P301S mutation only associated with frontotemporal dementia, supporting the notion that mouse modeling of nature development and progression of AD-related neurofibrillary tangles could be challenging without extensive genetic manipulations.

##### APOE KI mouse models

Apolipoprotein E (*APOE*) is a gene involved in the metabolism of lipids in the brain. In humans, the *APOE* gene is polymorphic with 3 alleles (*APOE2*, *APOE3*, and *APOE4*) of a frequency of 7%, 77%, and 15%, respectively [105]. Three types of APOE KI models were developed including targeted replacement APOE KI mouse models, Floxed APOE KI mouse models, and the APOE KI mouse models generated by the JAX lab.

**Targeted replacement APOE KI**

Through the targeted replacement, human APOE KI mice express human *APOE* alleles from the endogenous mouse *APOE* locus by homologous recombination approaches. It was found that in humanized APOE KI mice, APOE was primarily expressed in CNS glial cells. Levels of human APOE in the hippocampus and frontal cortex were similar between the APOE KI mice and non-demented human brain tissue, but the levels of APOE2 were higher than APOE3 and APOE4 levels in the blood. It was also found that cerebellar APOE levels were significantly higher than cerebral APOE levels [106]. There was no significant difference in plasma lipid and apolipoprotein levels in APOE3 KI and APOE4 KI mice, however APOE4 KI mice had about twice the amounts of cholesterol, APOE, and APOB-48 in their VLDL compared to APOE3 KI mice [107]. Therefore, it is important to note these varying levels of APOE isoforms across brain regions as well as differences in plasma lipid contents when characterizing AD-related pathological processes using these mouse models.

APOE4 KI mice had an increased accumulation of neuronal Aβ_42_ leading to mitochondrial changes, whereas Aβ_42_ levels in APOE3 KI mice were decreased between 1 and 4 months of age [108]. By crossing APOE KI mice and 5XFAD mice (EFAD mouse models), it was found that human Aβ_40_ and Aβ_42_ levels in female E3FAD and E4FAD mice were significantly higher compared to their respective male counterparts [109]. Moreover, APOE4 KI mice without 5xFAD background were observed to have increased levels of tau hyper-phosphorylation levels while APOE3 KI mice without 5xFAD background were found to have decreased phosphorylation levels between 1 and 4 months of age [108]. It was also found that compared to the E3FAD mice, the E4FAD mice demonstrated a faster deterioration of the blood-brain barrier (BBB), a reduced cerebral blood flow, and a greater degree of neuronal loss [109]. Furthermore, studies of PS19 mouse models in the background of human APOE KI or APOE^−/−^ reported a more dramatic degree of neurodegeneration and brain atrophy, as well as a higher level of insoluble tau formation in 9 months old APOE4/PS19 mice when compared to APOE3/PS19 mice, and APOE knockout in this case completely abolished tauopathy-related neurodegeneration [110]. In contrast, brain atrophy was not seen in 9.5 months old APOE4 mice with 5xFAD or APP/PS1 background, when significant amyloid burden was present at this age [111].

Female APOE4 KI mice were shown to have significant deficits in learning and memory that progressed with age as measured by the Morris water maze [112, 113]. These deficits were prevented by tau removal that were subsequently abolished by blocking the GABA signaling, suggesting APOE4-induced age- and tau-dependent cognitive impairments [112, 113]. It was also found that the hippocampal pathological effects of APOE4 were associated with impairments in spatial navigation [108]. The E4FAD mice developed hippocampal-associated memory deficits as measured by the Novel Object Recognition (NOR) test compared to E3FAD mice [109, 113], and APOE4 mice with or without 5xFAD background had a substantial drop in the nest construction scores compared to APOE3 counterparts as measured by daily activity tests regardless of Aβ pathology [109].

Because of APOE’s critical roles in AD pathogenesis, APOE KI mouse models have been widely used to investigate multiple disease processes other than the regulation of amyloid and tau pathologies, e.g. dysregulated brain phosphoinositol biphosphate (PIP_2_) homeostasis and cholesterol metabolism [114, 115]. Intriguingly, impaired cholesterol metabolism in oligodendrocytes with associated myelination deficits was reported using postmortem human brain samples, as well as APOE4 KI mice and human iPSC derived oligodendrocytes cells, and treatment with cyclodextrin to reduce intracellular cholesterol accumulation rescued white matter dysfunction and improved cognitive performance in APOE4 KI mice [116].

Human APOE mouse models have been used to study microglial dysfunction [117] and neuro-inflammation as well. For examples, it was described that in human APOE KI mouse models, LPS administration increased brain TNFα and IL6 levels in APOE4 mice [118], and APOE4 microglia released much higher levels of nitrite oxide (NO) than APOE3 microglia [119]. In E4FAD mouse models [120, 121], increased glial activities measured by IL1β levels exhibited negative effects on microglial morphology [122]. In the APOE4/PS19 mouse model, up-regulation of pro-inflammatory genes was observed whereas microglia in APOE^−/−^/PS19 mice remained homeostatic [110]. Moreover, depletion of microglia by PLX3397, or depletion of T-cells by neutralizing antibodies (anti-CD4 and CD8 antibodies) in APOE4/PS19 mice prevented the infiltration of T cells into the CNS and rescued neurodegeneration, implicating a role of innate and adaptive immune dysfunction in tauopathy-mediated neurodegeneration [111].

**Floxed APOE KI**

Similarly to the Targeted Replacement model, the Floxed APOE KI model aims to better investigate the function of various APOE isoforms in AD. The Floxed model was created with the coding region of mouse *APOE* gene was replaced with corresponding human *APOE* sequence and flanked by loxP sites [123]. This allows cell type-specific control of gene expression. These mouse models can then be crossed with mouse models of amyloidosis or tauopathy to observe how various APOE isoforms affect AD-related pathological features.

The APOE protein was found in the astrocytes but not in reactive Iba-1 positive microglia surrounding amyloid plaques. By crossing APP/PS1 transgenic mice with various Floxed APOE KI mice, it was demonstrated that the APOE4 mice had a greater effect on amyloid accumulation [123, 124]. It was also found that APOE deletion in hepatocytes did not affect brain APOE levels but a decrease in plasma APOE levels and changes in plasma lipid profiles were noted [124].

In Floxed APOE KI crossed with PS19 mice at 10-month of age, PS19-E4 mice demonstrated a higher degree of neurodegeneration compared to PS19-E3 mice, including increased hippocampal volume loss and tauopathy, myelin abnormalities and gliosis [125, 126]. Neuronal knockout of APOE4 led to a dramatic reduction in tau pathology and neurodegeneration relative to mice that did not have their neuronal APOE4 removed, whereas no significant reduction in tau pathology or neurodegeneration was noted between APOE3 mice with neuronal APOE removed and mice that did not [125, 126]. It was speculated that APOE4 plays a critical role in the promoting the development of major AD-related pathologies and its removal in neurons can mitigate APOE4-driven tauopathy and neurodegeneration [125, 126].

**APOE KI:** **JAX**

Similarly to the other APOE KI models, APOE KI mouse models developed by the JAX Lab, expressed humanized *APOE* alleles from endogenous *APOE* locus by replacing mouse *APOE* exons 2, 3, and most of 4 with human *APOE* sequence (provided by the Alzforum). At 2 and 12 months of age, locomotor activity and motor coordination tested by open field and rotarod tests, respectively, as well as working memory by the Y-maze tests were similar between APOE4 KI and control mice with an age-dependent decline (provided by Alzforum). It was found that female APOE4 KI JAX mice had lower plasma Aβ_42_ levels and a decreased Aβ_42_/_40_ ratio which would suggest higher levels of aggregate-prone Aβ_42_ in the brain, compared to male APOE4 KI mice. However, there were no differences seen between APOE4 and APOE3 KI mice. In addition, plasma Aβ_40_ levels did not differ regardless of APOE genotype or sex [127].

In summary, humanized APOE KI mouse models were established to study the functional roles of human APOE genotypes in AD-related processes without the confounding effects of murine APOE. However, human APOE KI mouse models often crossed with AD transgenic mouse models (e.g. 5xFAD or PS19) in order to study human AD-related pathological changes. Many AD changes other than amyloid and tau pathology have been reported in these mouse models (APOE4/5xFAD or APOE4/PS19) such as dysfunction in lipid metabolism and immune systems, highlighting the importance of incorporating APOE4 genotype into the studies of AD pathological processes. Moreover, sex differences as well as sex-dimorphic responses to genetic or pharmacological manipulations in human APOE mouse models have been reported, implicating the interaction between sex and APOE in AD pathogenesis [112, 113, 128]. Overall, selecting appropriate humanized APOE mouse models with or without AD transgenic background taking into account of the impact of biological variables such as age, sex and human APOE genotypes on disease processes will help guide a better understanding of multifaceted disease mechanisms as well as precision-medicine directed development of therapeutic strategies.

##### TREM2 MODELS (KO/KI and Tg)

TREM2 is a cell-surface receptor and a transmembrane protein that is crucial for coordinating cellular immune responses. TREM2 signals through an adaptor protein DAP12, which is associated with the downstream tyrosine kinase SYK [129]. The Collona line was generated by deleting exons 3 and 4 of the *TREM2* gene. While TREM2^−/−^ mice did not exhibit a clear neurological phenotype besides microglial deficiencies [130], they were associated with development of bone diseases and FTD [131]. The R47H missense variant of TREM2 has been associated with impaired TREM2 function [132, 133].

**TREM2**^**−/−**^ **mouse models**

TREM2^−/−^ mice demonstrated an increased production of TNF-α and a slight increase in production of IL-6 in bone marrow-derived macrophages after treatment of LPS, zymosan, and CpG [134]. It was also found in vitro that TREM2 responded to lipidic components of myelin, indicating a role in detecting myelin damage [130]. When mice were fed with a copper chelator cuprizone (CPZ), causing apoptosis of mature oligodendrocytes (ODCs), TREM2^−/−^ mice exhibited an age-related decrease in microglial numbers, microglial size, and transcriptional responses for myelin repair. These impairments led to a prolonged microgliosis as well as an impaired remyelination and repopulation of ODCs. TREM2^−/−^ mice fed with CPZ showed a decreased performance on motor coordination tests such as the rotarod tests at 12 months of age but did not show any significant differences in locomotor activity, balance, or grip strength when compared to wt controls [135]. TREM2 was found to play a leading role in conversion of disease-associated microglia (DAM), with TREM2-null microglia being able to clear myelin debris but not myelin-associated cholesterol. The CPZ-challenged TREM2^−/−^ mice had increased levels of APP^+^ puncta and neurofilament-light chain (NFL) expression in the hippocampus and corpus collosum regions [136].

As a microglial receptor with immunoglobulin-like ectodomain binding domains, TREM2 can bind to multiple ligands such as APOE and phospholipids [136, 137]. In the TREM2^−/−^ x 5xFAD mouse model, a significantly higher amount of amyloid accumulation was seen in hippocampal regions when compared to TREM2 wt counterparts. However, while microglia behaved similarly to wt microglia, it failed to exhibit an upregulated transcriptomic response to amyloidosis, indicating that TREM2 is necessary for microglia activation in response to amyloid. The IBA1 positivity was reduced in TREM2^−/−^ x 5xFAD mice in comparison to 5xFAD only mice, especially around amyloid plaques [132, 133]. It was suggested that TREM2 may act as a costimulatory molecule that helped sustain microglia during amyloidosis when colony stimulating factor (CSF)-1 levels were reduced.

Moreover, TREM2 was found to detect damage-associated lipid patterns associated with neurodegeneration, sustaining microglial responses to Aβ accumulation [133]. In addition, an absence of TREM2 or APOE was associated with impaired cholesterol transport and metabolism in microglia [136]. In an APP/PS1ΔE9 mouse model expressing human APOE3 or APOE4, TREM2-deficiency increased plaque growth in the early stages of amyloidosis with a decreased microglial response, without affecting an overall level of plaque deposition. A significant decrease in APOE4 mRNA and protein expression in plaque-associated microglia was observed in TREM2 KO mice, with no changes in plaque-associated APOE protein expression in APP/APOE3 mice. Additionally, TREM2 deficiency increased the plaque growth in APP/APOE3 mice, but not in APP/APOE4 mice. The number of differentially expressed genes were found to be over two times higher in APP/APOE4 *versus* APP/APOE3 compared to APP/APOE4/TREM2^−/−^*versus* APP/APOE3/TREM2^−/−^, despite TREM2^−/−^ mice having a similar amount of neurodegeneration as their WT counterparts likely due to an impaired immune response in TREM2^−/−^ mice [138].

Interestingly, when TREM2^−/−^ mice were crossed with PS19 (TREM2^−/−^ PS19), decreases in neurodegeneration and synaptic degeneration were observed in the entorhinal and piriform cortex of TREM2^−/−^ PS19 mice when compared to TREM2^+/+^ PS19. Additionally, it was found that in 9-month-old mice, there were no significant differences in p-tau levels or tau solubility between TREM2^−/−^ PS and TREM2^+/+^ PS mice. TREM2^−/−^ PS mice also demonstrated significantly reduced microgliosis and fewer microglia with a more ramified shape. These mice also showed lower levels of microglia-activating transcripts such as *apoe* and *cst7*, decreased levels in several inflammatory markers, and reduced astrogliosis. These results altogether suggested that a loss of TREM2 function reduced microglial responses to tau pathology as well, thus reducing neurodegeneration [139].

**hTREM2 KI mouse models**

There have been several human TREM2 R47H mouse models developed in the field. The Song et al. generated TREM2 common variant (TREM2^CV^) and TREM2 R47H variant (TREM2^R47H^) Tg mouse lines backcrossing with mouse TREM2^−/−^ to obtain normal TREM2 levels and then crossed with 5xFAD background to study functions of TREM2 R47H variant in the presence of human AD pathology [132]. It was found that TREM2 R47H variant impaired TREM2 function including lipid sensing and DAM responses to amyloid [132, 133]. In addition, the TREM2 R47H variant was found to impair soluble TREM2 cell-surface interactions, with a decreased TREM2 shedding noted on neurons and around amyloid plaques in the TREM2 R47H KI x 5xFAD mouse model [132]. The IBA1 positivity was reduced in TREM2 R47H KI x 5xFAD in comparison to 5xFAD only mice, similarly to what was seen in TREM2^−/−^ x 5xFAD mouse model [132, 133]. Moreover, the effects of TREM2 R47H variant on tauopathy were described using the same TREM2^CV^ and TREM2^R47H^ mouse lines crossing with PS19 mice [140]. The PS19-TREM2^R47H^ mice developed less brain atrophy and synaptic loss with diminished microglial reactivity and phagocytosis when compared to PS19-TREM2^CV^ mice. These findings along with the observations from TREM2^−/−^ PS19 mice support the distinct effects of TREM2 on microglial function in the presence of amyloid pathology *versus* tauopathy, adding layers of complexity about TREM2 and its role in AD pathogenesis.

More recently, the TREM2-DAP12 and downstream signaling pathways such as activation of a protein tyrosine kinases SYK, were found to be impaired with TREM2 R47H, and that antibodies against CLEC7A directly activated SYK could rescue some phenotypes in TREM2 R47H mice [141]. Furthermore, a defective mTOR pathway with autophagy dysfunction in the TREM2-DAP12 signaling pathways can be offset through administration of cyclocreatine, thus restoring microglial recruitment around amyloid plaques [129]. Recently, a study demonstrated that up-regulation of TREM2 by AL002c reduced plaque burden, decreased neurite dystrophy, rescued abnormal behaviors such as risk-taking and exploratory drive, and attenuated microglial inflammatory responses [142].

Besides the hTREM2 KI mouse lines developed by Colonna and others, several lines of TREM2 R47H KI mouse models were generated as well. For example, Cheng-Hathaway et al. generated a heterozygous TREM2 R47H mouse model using CRISPR/cas9-mediated insertion of human *TREM2*^*R47H*^ coding region into mouse *TREM2* gene with APP/PS1-21 background (APPPS1-21/TREM2^+/R47H^) [143]. The TREM2 R47H variant in these mouse model led to attenuated microglial responses to amyloid with reduced amounts of dense-core plaques and increased neurite dystrophy. Furthermore, Xiang et al. using the CRISPR/cas9 approaches generated wt, heterozygous and homozygous TREM2 R47H KI mouse models [144]. Intriguingly, an atypical cryptic splicing of mouse TREM2 R47H was noted in these mouse models raising the concerns of previously described phenotypes in KI mouse models generated by similar CRISPR gene editing of mouse *TREM2* gene may artificially introduced *TREM2* haploinsufficiency phenotypes that were not commonly seen in human TREM2 R47H condition [144]. Alternatively, a TREM2 R47H normal splicing site (TREM2 R47H^NSS^) mouse model was generated to characterize the function of hTREM2 R47H variant with comparable expression levels of TREM2 to wt mice without the impact of cryptic splicing [145]. In 5xFAD background, TREM2 R47H^NSS^ mice with cuprizone-induced neuro-inflammation demonstrated age-dependent impairments in microglial interaction with plaques (only seen at 4-month of age), as well as LTP deficits and synaptic loss (seen at 12-month of age) [145].

Recently, the JAX lab developed a few mouse models such as the LOAD1 mouse model carrying human APOE4 and TREM2 R47H variant (double KI homozygous) [146] and the LOAD2 mouse model carrying APOE4/TREM2 R47H/hAPP KI gene expression (triple homozygous) [147] as part of the MODEL-AD consortium. It was reported that there were no amyloid plaque or other AD hallmark changes observed, nor any cognitive deficits detected in the LOAD1 mouse models even at 24 months of age. However, the reduction in brain TREM2 protein levels and changes in circulating cytokine levels, in addition to regional changes in glycolysis and vascular perfusion were noted when compared to C57/B6 controls [146]. Sex differences were described with female LOAD1 mice showing increased risks of mortality than males. In addition, glycolysis in female mice was significantly altered at 4-month of age and persisted till 12-month of age, whereas males demonstrated a transient hypoglycolytic phenotype at 8-month of age then normalize in later ages [146]. On the other hand, after 16 months of high fat diet (HFD) treatment, the LOAD2 mice demonstrated neuronal loss, elevated brain Aβ_42_, and behavioral deficits assessed by some touchscreen cognitive tasks, in addition to brain volume loss and neurovascular uncoupling [147].

Currently, several novel mouse strains have been generated through the MODEL-AD consortium with various newly identified LOAD risk genes further introduced in the background of LOAD1 (APOE4/TREMR47H KI) such as PLCγ2 M28L, MTHFR C677T and SORL1 A528T. The phenotype characterization of these mouse models is currently ongoing with one recent publication describing LOAD1.PLCγ2 M28L and LOAD1.MTHFR C677T mouse models [148]. It was reported that these mice after HFD treatment demonstrated glucose and cholesterol increase with some changes in microglial density, brain regional glucose and vascular perfusion only seen in LOAD1.PLCγ2 M28L mice not in LOAD1 alone or LOAD1.MTHFR C677T mouse models [148]. Together, these studies and effort of developing new models hopefully could lead to a better understanding of functional roles of new LOAD risk factors, in addition to other environmental factors (e.g. HFD) in AD pathogenesis.

**BAC hTREM2 transgenic mice**

While extensive studies in the field have been focused on using TREM2 KO or loss-of-function variant mouse models to understand its functional roles in AD, there were TREM2 Tg mouse models developed to determine the effects of TREM2 over-expression under genomic regulation through a bacterial artificial chromosome (BAC) transgenic approach [149, 150]. In BAC human TREM2 Tg mice with 5xFAD background, there were reduced amyloid plaques with associated gene signature changes including dampened damage-associated microglial gene expression and up-regulated neuronal gene expression. Furthermore, cognitive performance was improved in BAC hTREM2 Tg x 5xFAD mice compared to 5xFAD alone mice with increased phagocytic microglia and reduced neurite dystrophy [151]. Therefore, these findings implicate the beneficial potential of elevating TREM2 expression in modulating microglial function in AD.

In summary, various TREM2 mouse models have been versatile in illuminating the role of TREM2 in the AD brains and revealing novel pathways for microgliosis. Promising treatments designed to target TREM2 and its related signaling pathways have been actively explored in the AD field with the knowledge gained from these mouse models. However, there are growing concerns about the applicability of these mouse models into studying microglia function in AD. Notably, there are differences in mouse and human immune genes, as well as distinct microglial aging processes between mouse and human [152]. Moreover, the opposite phenotypes observed in TREM2 KO and TREM2 R47H KI mouse models in the presence of amyloid pathology [132, 133] *versus* tauopathy [139, 140] further implicate the complexity of TREM2 function in AD pathogenesis.

#### Other mouse models

##### SAMP8 mouse model

Senescence-accelerated mice (SAM) are a type of accelerated aging model that was produced through selective breeding of AKR/J strain mice. Through selective breeding, many senescence-accelerated mouse prone (SAMP) and senescence-accelerated mouse resistant mouse strains were produced. The SAMP8 sub-strain was shown to be a viable model to study AD with well characterized age-related neuropathological changes, as well as learning and memory deficits [153].

SAMP8 mice displayed age-related Aβ deposition and hyperphosphorylation of neurofibrillary tangles. Aβ was found in the hippocampus as early as 6 months of age and in the cerebral cortex as early as 9 months of age [154]. NFTs have been found in the cerebral cortex at 5 months of age and hippocampus at 3 months of age [154]. In addition, SAMP8 mice had elevated biomarkers of oxidative stress [155], inflammation [156], mitochondrial [157], and blood-brain barrier dysfunction [158, 159]. Neuronal loss was noted in the hippocampus, cerebral cortex, and forebrain regions; dendrite spine loss was noted in the hippocampus, brain stem, and spinal cord; microgliosis noted in the cerebral cortex and hippocampus; and astrogliosis noted in the brainstem, spinal cord, as well as cerebral and cerebellar white matter [154].

SAMP8 mice demonstrated memory and learning impairments measured by various behavioral tests such as passive avoidance tasks and Morris water maze tasks [160]. As early as 2 months of age, SAMP8 mice showed impairments in the acquisition of passive avoidance response and became increasingly impaired over times with significant impairments noted at 12 months of age. In addition, SAMP8 mice demonstrated spatial learning impairments measured by the Morris water maze task as early as 2 months of age. The circadian rhythm was found to be abnormal as well [160].

In summary, the SAMP8 mouse model provides insights into AD-related pathology, as well as learning and cognitive deficits. One noticeable shortcoming of the SAMP8 mice is their shortened lifespans. The average lifespan of SAMP8 mice was about 10–12 months while the median life expectancy was 9.7 months [161].

##### Seeding models

In recent years, it has been found that cell-to-cell transmission of protein aggregates plays a role in the progression of neurodegenerative disorders like AD. Prion-like seeding is described as an accelerated nucleation-dependent polymerization process. Previous studies using brain extracts from AD human patients or APP-transgenic mice accelerated progression and severity of amyloid aggregation and deposit in various APP transgenic models [162] such as Tg2576 [163]. It has been speculated that Aβ seeding induced the spreading and dissemination of amyloid pathology through endo-lysosomal and vascular/perivascular involvement [162]. Among different Aβ seeding experimental paradigms, one variable is the selection of different Aβ seeds [164]. Evidence suggest that Aβ oligomers were important in activating aggregation in early phase of seeding process [162, 165]. Furthermore, the cross-seeding of Aβ including homologous cross-seeding within different amyloid species as well as heterologous cross-seeding with other pathology such as α-synuclein has been a point of interest [166]. For example, in 5xFAD mice, inoculation of α-synuclein preformed fibrils in 5xFAD mice led to accelerated seeding and spread of α-synuclein and tau, as well as exacerbated AD pathology including increased amyloid plaque burden, tau hyper-phosphorylation and neurite dystrophy when compared to wt mice inoculated with α-synuclein seeds [167].

On the other hand, seeding has been shown to accelerate pathology induced by the misfolded tau species as well, initially in mouse models over-expressing tau and later in non-transgenic mice [168]. It was found that inoculation of human AD brain-derived tau fibrils (AD-tau) were effective in seeding and spreading of tau pathology in vivo in aged non-Tg mice [168]. Interestingly, it was also found that in a mouse model that expressed an equal ratio of 3R and 4R human tau isoforms (6hTau mice), inoculation of different strains of tau seeds derived from different tauopathy human brains such as AD (mixed 3R and 4R tau), Pick’s disease (mostly 3R tau), progressive supranuclear palsy (mostly 4R tau) and corticobasal degeneration (mostly 4R tau) led to distinct conformation-dependent cell-type specific transmission of tauopathy without significant cross-seeding of non-corresponding tau isoforms in vivo [169].

In summary, we focused on several commonly used AD mouse models (Tables 1 and 2), as well as a few newly developed AD mouse models in the field. However, it should be noted that there is ongoing effort to develop new mouse models to better understand multi-faceted heterogeneous disease mechanisms such as the contribution of endo-lysosomal dysfunction, lipid metabolism, vascular disease and immune response system to AD. For example, the MODEL-AD consortium has been generating AD mouse models expressing newly identified LOAD risk variants with some described in above section under the “TREM2 mouse models (KO/KI and Tg)”, characterized by deep phenotyping approaches including traditional neuropathological and behavior measures as well as complementary Omics approaches to profile gene expression pattern of mouse brains. The data have been made publicly available via the AD Knowledge Portal (https://adknowledgeportal.synapse.org/Explore/Programs/DetailsPage?Program=MODEL-AD) with transcriptomics data of some mouse models curated and analyzed in following section.

### Transcriptomic profiling of molecular signatures of AD mouse models

We curated total 11 publicly available transcriptomic data including datasets generated from microglia derived from AD mouse brains as well as datasets from bulk brain tissue of various AD mouse models (Table 3) [99, 133, 170–174]. Data processing was performed as previously described [175] with gene expression level quantified by FeatureCounts (v1.6.3) [176] and gene read count normalized using trimmed mean of the M-values normalization methods [177] to adjust for sequencing library size difference. Differential gene expression between different comparison groups was predicted by a linear model analysis using R/Bioconductor package limma [178, 179]. A gene was considered significant when fold change ≥ 1.2 and adjusted *p* value ≤ 0.5. Functional annotation of the DEGs was assessed by enrichment for the MSigDB gene ontology (GO) and pathway collections using the hypergeometric test. To adjust for multiple tests in either differential expression signatures or GO/pathway enrichments in each dataset, the Benjamini-Hochberg false discovery rate (FDR) method was employed [180].

**Table 3:** Information about publicly available mouse transcriptomic datasets used for the analysis in this study including mouse AD gene signatures derived from microglia of AD mouse models and AD mouse brain bulk tissue transcriptomics datasets with details including mouse cohort labels (used in the portal and figures), study traits, brain regions (or cell types) used in the study, accession ID# and references

	Mouse Cohort Label	Trait	Brain Region (or Cell Type)	Accession ID#	Ref
1	5xFAD	Trem2-/- in WT and 5XFAD mice	CTX/HPC (microglia)	GSE65067	[133]
2	TREM2_KO	WT, TREM2+/+, TREM2-/- and TREM2-/- mice	CTX (microglia)	GSE107293	[174]
3	APP_SAA	WT, App-SAA [Swedish (KM670/671NL), Arctic (E693G) and Austrian (T712I)]. App-SAA: methoxy-positive and methoxy-negative fractions; WT: methoxy-negative	CTX/HPC (microglia)	GSE158153	[99]
4		WT and App-SAA. App-SAA: HOM and Het; WT		GSE158152	
5	TREM2-/-	WT and TREM2-/-, Basal or after LPS administration	HPC (mix cell population)	Original publication (Table S1 and S2)	[171]
6	Tg2576	Tg2576 Swedish APP (KM670/671NL)	CTX (mix cell population)	Original publication	[173]
7	TgCNRD8	TgCRND8 Swedish plus Indiana APP (KM670/671NL + V717F)	CTX (mix cell population)	Original publication	[173]
8	3xTg	WT, 3xTg-AD (APP Swedish, MAPT P301L, and PSEN1 M146V)	RB CTX/HPC (mix cell population)	syn22964719	[172]
9	5xFAD	5xFAD, WT	RB HPC/CTX (mix cell population)	syn18637070	[172]
10	hAbeta_KI	WT, human AbetaKI	RB HPC (mix cell population)	syn18670930	[172]
11	5xFAD	5xFAD, WT	RB (mix cell population)	syn23638028	[170]
12	Apoe4/Trem2_R47H	WT and APOE4.Trem2R47H: Replace the exons 2, 3 and most of exon 4 from the mice to the human APOE4 gene sequence, and a KI point mutation in Trem2 (R47H), along with 2 other silent mutations	RB (mix cell population)	syn18637029	[170]

We first evaluated whether specific molecular changes in AD mouse models are reminiscent of molecular signatures of human AD brains by performing intersection studies between the DEGs of available mouse brain transcriptomic signatures and a collection of published human AD brain transcriptomic signatures including data from different AD-related clinical/pathological traits and different brain regions (Table 4) [181–191]. In addition, we performed intersection studies between the DEGs of available mouse microglial transcriptomic signatures and a collection of published transcriptomic signatures from the single-cell RNA-sequencing (scRNA-seq.) studies of microglia or myeloid cells derived from human AD brains (Table 4) [192, 193]. For example, the Mount Sinai Brain Bank (MSBB) AD study contains signatures from 4 different brain regions (Brodmann area 10 (BM10) frontal pole, Brodmann area 22 (BM22) superior temporal gyrus, Brodmann area 36 (BM36) parahippocampal gyrus, and Brodmann area 44 (BM44) inferior frontal gyrus) with respect to 4 AD related semi-quantitative traits (clinical dementia rating (CDR), mean plaque density (PlaqueMean), CERAD, and Braak score) [189, 191]. The comparisons of MSBB signatures were performed between sample groups defined for each trait. The *p* value significance of the intersections was computed by the hypergeometric test. The data portal used to analyze individual mouse and human brain transcriptomic datasets including DEGs, GO/pathway (heatmaps, bar plots and Sankey plots), as well as mouse and human signature overlay studies can be accessed at https://rstudio-connect.hpc.mssm.edu/admouse/.

**Table 4:** Information about publicly available human transcriptomic datasets for the analysis in this study including human AD gene signatures derived from brain bulk tissue transcriptomics datasets as well as scRNA-seq. of microglia from human AD brain with details including cohort labels (used in the portal and figures), data information, brain regions, cell types and references

Human Cohort Label	Data Information	Brain Region	Cell type	Ref
Allen (Mayo)	Brain transcriptomes from patients with AD, progressive supranuclear palsy (a primary tauopathy), and control subjects in the Mayo clinic cohort.	Cerebellum region, temporal cortex	Mixed cell population	[181]
Avramopoulos	Microarray: identify genes involved in normal aging and genes involved in AD. RNA extracted from the temporal lobe of 22 late onset AD and 23 control brain donors.	Temporal lobe	Mixed cell population	[182]
Blalock	Microarray: nine control and 22 AD subjects of varying severity.	Hippocampus	Mixed cell population	[183]
Colangelo	Microarray: Hippocampal regions was pooled from six controls and six AD subjects.	Hippocampus CA1 region	Mixed cell population	[184]
Liang	Microarray (Affymetrix Human Genome U133 Plus 2.0 microarrays): Normal cortical neurons collected with laser capture microdissection. Overall regional analyses consisted of 11 to 13 control subjects and 10 to 23 AD subjects that varies from different brain regions.	6 brain regions: (1) entorhinal cortex, (2) hippocampus, (3) medial temporal gyrus, (4) posterior cingulate, (5) superior frontal gyrus, and (6) primary visual cortex.	Mixed cell population	[185]
Miller	Microarray (Illumina HumanHT-12 V3.0 expression beadchip): 32 control, 31 AD.	Hippocampus CA1 and CA3 regions	Mixed cell population	[186]
Mostafavi (ROSMAP)	RNA-Seq from the ROSMAP cohort (total *n* = 478). Parameters such as signature correlated with B amyloid, cognitive decline, clinical diagnostic of AD and AD pathology were considered.	Dorsal lateral frontal cortex (DLPFC)	Mixed cell population	[187]
Satoh	RNA-Seq Data Mining	Frontal cortex	Mixed cell population	[188]
Webster	Analyzed samples with a confirmed pathologic diagnosis of late-onset Alzheimer disease (LOAD; final *n* = 188 controls, 176 cases. AD signature).	Temporal cortex	Mixed cell population	[190]
Zhang (HBTRC)	Postmortem brain tissues from LOAD patients and nondemented subjects in the Harvard brain tissue resource center (HBTRC). Analysis of parametrs such as atrophy and Braak staging.	Cerebellum, prefrontal cortex	Mixed cell population	[189]
Wang (MSBB)	RNA-seq of four cortical areas from 364 donors in the Mount Sinai Brain Bank (MSBB) cohort with varying degrees of severity regarding 4 cognitive/pathological phenotypes. Study investigated parameters such as CERAD Definite AD vs NL or AD vs NL; Plaque Mean Normal, Mild and Severe. CDR Demented vs MCI vs nondemented. Braak score AD vs NL.	Brodmann area 10 frontal pole (BM10-FP), Brodmann area 22 superior temporal gyrus (BM22-STG), Brodmann area 36 parahippocampal gyrus (BM36-PHG), and Brodmann area 44 inferior frontal gyrus (BM44-IFG)	Mixed cell population	[191]
Olah (ROSMAP)	Single cell RNA-seq of human microglia obtained at autopsy from 9 AD and 4 MCI cases.	Dorsolateral prefrontal cortex (DLPFC)	Microglia	[192]
Lee	Single cell RNA-seq of myeloid cells from postmortem brain specimens of two cohorts (*n* = 137 and 1,470) with varying degrees of AD neuropathology.	Prefrontal cortex	Myeloid	medRxiv.2023.10.25.23297558
Sun (ROSMAP)	Single-nucleus microglial transcriptomes and epigenomes of 194,000 nuclei across 443 human subjects and diverse Alzheimer’s disease (AD) pathological phenotypes.	Prefrontal cortex, hippocampus, mid-temporal cortex, angular gyrus, entorhinal cortex, and thalamus	Microglia	[193]

As shown in Fig. 2, mouse models that showed most significant overlapping of molecular signatures with human AD signatures derived from Brodmann area 36 (BM36) para-hippocampal gyrus brain region of Mount Sinai Brain (MSBB) AD study [189, 191] were 5xFAD (both JAX and UCI models with both down-regulated and up-regulated DEGs) as well as TgCRND8 mice (mostly up-regulated DEGs). There were some overlapping with TREM2 mouse models including TREM2 KO and APOE4/TREM2 R47H (mostly down-regulated DEGs) as well. The GO pathway enrichment analysis indicated changes in several AD-related pathways and functions in these mouse models, such as up-regulation of immune system responses as well as down-regulation of synaptic activities (https://rstudio-connect.hpc.mssm.edu/admouse/).

Intriguingly, we have noted that different mouse models represent different aspects of AD molecular signatures of human AD brains. For example, 5xFAD mouse models demonstrated molecular signatures of amyloid fibril formation, binding to and responses to amyloid whereas APOE4 and TREM2 R47H with 5xFAD background, as well as TgCRND8 mice in addition to some 5xFAD model showed molecular signatures relevant to amyloid clearance (Fig. 3A). On the other hand, a few mouse models including 5xFAD, TgCRND8, TREM2 KO as well as APOE4 and TREM2 R47H with 5xFAD background manifested molecular signatures of neuroinflammation including neuroinflammatory responses, antigen processing and presentation as well as adaptive immune responses seen in human AD brains (Fig. 3B). It has been noted that 5xFAD mouse models demonstrated a significant overlapping of human AD molecular signatures other than amyloid and tau pathology such as neuroinflammation, endo-lysosomal function and synapse formation, supporting the notion that this model showed most significant overlapping with human AD molecular signature and is probably a preferable mouse model to study certain AD processes such as neuro-inflammation and endo-lysosomal dysfunction. However, it is speculated that 5xFAD mouse models had most sophisticated sequencing analyses so far such as different ages, different brain regions, male *versus* female, as well as different datasets from mouse models generated from different sources which allowed deep phenotyping of molecular signatures of these mouse models in comparison to human brain datasets. Again, these findings further highlight the importance of future Omics studies of AD mouse models taking in considerations of variables (age, APOE genotypes, sex and brain regions) for better understanding how well aligned with human disease signatures. It should be noted that no significant overlapping between mouse and human molecular signature regarding tau pathology even with transcriptomic dataset generated from 3xTg-AD mouse brains. It is possible that limited transcriptomic data available from tauopathy models such as PS19 and tau KI mice may contribute to the lack of representation of tau pathology in AD mouse models. Future studies are needed to determine if molecular signatures of tauopathy mouse models (transgenic and/or KI models) could represent some aspects of human tau pathology during disease development and progression.

Furthermore, when we overlaid mouse microglia signatures with transcriptomic signatures derived from microglia or myeloid cells through scRNA-seq analysis of human AD brains [192, 193] (Tables 3 and 4), the most significant overlapping with human AD brain microglial/myeloid molecular signatures we observed were microglia signatures of TREM2^−/−^ mice in 5xFAD background as well as microglial signature from the APP_SAA mouse models (Figs. S1 and S2). Interestingly, the disease processes with significant overlaps are involved in neuroinflammation similarly to what we have seen with bulk brain tissue transcriptomic overlay studies (Fig. 3B), such as activation of immune responses, chemotaxis and circulatory system development (Fig. S1). In addition, some overlapping in protein/lipid metabolism have been noted such as lipid metabolic process, protein targeting, as well as protein catabolic and proteolytic processes (Fig. S2). These studies highlight the strengths of utilizing scRNA-seq. datasets in better characterizing molecular phenotypes of mouse models and matching with human AD brain molecular signatures.

In summary, we provided an overview of a few commonly used AD mouse models including transgenic and knock-in/knockout models in this review with the advantages and limitations of each model discussed along with age-related sex-specific phenotypic features summarized in Tables 1 and 2. More importantly, we analyzed the omics data from available mouse models (Table 3) to categorize molecular signatures reminiscent of human AD brain changes (Table 4) with data portal published at https://rstudio-connect.hpc.mssm.edu/admouse/ and described in Figs. 2, 3, S1 and S2). Our approaches highlight the importance of incorporating multi-omics analyses of mouse models into phenotypic characterization and posit a starting point for future research to compare brain molecular signatures of newly developed mouse models with human AD brain signatures with the hope to guide next steps of determination and selection of most suitable models for relevant research questions in AD field.

## Future directions

Novel technologies have been developed in a fast-growing field like AD research. For example, effort has been devoted to incorporating human cell model systems, such as human iPSC-derived brain cells like neuronal precursor cells and microglia [194, 195], as well as human brain organoids [196] into various mouse models to generate human and mouse chimeric systems (Fig. 1) with the hope to better understand the nature of human brain cells in aging and diseased conditions in vivo. While some of these human mouse chimeric systems manifested disease-like phenotypes [197], questions such as utilizing immunocompromised mouse models when studying the immune responses related to AD pathogenesis remain to be addressed with further characterization. Moreover, there are needs in the field to develop more diverse mouse models for the goals of studying heterogeneous mechanisms of AD that are associated with vascular disease, immune dysfunction, metabolic dyshomeostasis, as well as understanding the impact of environmental factors among others, individually or in combination with AD-associated genetic risk factors. On the other hand, new information obtained from next-generation multi-omics datasets (e.g., scRNA-seq. profiles) of human and mouse samples could guide the development and identification of novel risk variants and markers of AD leading to next-generation in vivo modeling of AD.

### Supplementary Information

**Additional file 1: Supplemental Figure 1.** Comparison of gene ontology (GO)/pathways between the AD mouse microglial datasets and human AD microglial datasets. Sanky network plots show the commonly shared GO/pathways between mouse AD gene signatures derived from microglia of AD mouse models (left) and human AD gene signatures derived from scRNA-seq. studies of AD human brain microglia (right). Each node represents a gene signature or a GO/pathway term. Each link colored based of individual GO/pathway term represents a significant overlap between mouse and human gene signatures. Commonly shared GO/pathways involved in neuroinflammation and immune responses in AD.

**Additional file 2: Supplemental Figure 2.** Comparison of gene ontology (GO)/pathways between the AD mouse microglial datasets and human AD microglial datasets. Sanky network plots show the commonly shared GO/pathways between mouse AD gene signatures derived from microglia of AD mouse models (left) and human AD gene signatures derived from scRNA-seq. studies of AD human brain microglia (right). Each node represents a gene signature or a GO/pathway term. Each link colored based of individual GO/pathway term represents a significant overlap between mouse and human gene signatures. Commonly shared GO/pathways involved in protein and lipid metabolism in AD.

## Data Availability

The datasets downloaded, generated and/or analyzed during the current study include publicly available datasets downloaded from the GEO and the AMP-AD portal with data information provided in the Tables (Tables 3 and 4). The data portal used to analyze individual mouse and human brain transcriptomic datasets including DEGs, GO/pathway (heatmaps, bar plots and Sankey plots), as well as mouse and human signature overlay has been published and made publicly accessible at https://rstudio-connect.hpc.mssm.edu/admouse/.

## Abbreviations

AD: Alzheimer’s Disease
Aβ: Amyloid beta
APP: Amyloid precursor protein
APOB: Apolipoprotein B
APOE: Apolipoprotein E
APOE4: Apolipoprotein E4
BAC: Bacterial artificial chromosome
BBB: Blood-brain barrier
BDNF: Brain derived neurotrophic factor
BM10: Brodmann Area 10
BM22: Brodmann Area 22
BM36: Brodmann Area 36
BM44: Brodmann Area 44
CNS: Central Nervous System
CDR: Clinical Dementia Rating
CERAD: Consortium to Establish a Registry for Alzheimer’s Disease
CPZ: Cuprizone
CPM: Count per Million
CRISPR: Clustered Regularly Interspaced Short Palindromic Repeats
CSF-1: Colony Stimulating Factor-1
DEGs: Differentially expressed genes
DAM: Disease Associated Microglia
dKI: Double Knock-In
EEG: Electroencephalogram
ER: Endoplasmic Reticulum
FAD: Familial AD
FDR: False Discovery Rate
Fgf14: Fibroblast growth factor 14
FLP: Flippase
FPKM: Fragment Per Kilobase of transcript per Million
FTD: Frontotemporal dementia
GO: Gene Ontology
GSEA: Gene set enrichment analysis
HEXB: Hexosaminidase Subunit Beta
HFD: High fat diet
iPSC: Induced Pluripotent Stem Cells
IL6: Interleukin-6
KI: Knock-In
KO: Knock-Out
LOAD: Late-onset AD
LPS: Lipopolysaccharide
LTP: Long-term Potentiation
LAMP2: Lysosomal Associated Membrane Protein-2
MRI: Magnetic Resonance Imaging
MRS: Magnetic Resonance Spectroscopy
mTOR: Mammalian target of rapamycin
MAPT: Microtubule-Associated Protein Tau
MSBB: Mount Sinai Brain Bank
NAA: N-acetylaspartate
NAA/Cr: N-acetylaspartate/Creatinine
NFTs: Neurofibrillary Tangles
NFL: Neurofilament Light Chain
Nrf2: NF-E2 related factor 2
NO: Nitrite Oxide
NREM: Non-rapid eye movement
NOR: Novel Object Recognition
ODC: Oligodendrocytes
PPF: Paired-pulse facilitation
PAC: Phase amplitude coupling
PLD3: Phospholipase D Family Member 3
PFC: Prefrontal Cortex
PS1: Presenilin 1
PrP: Prion protein
H NMR: Proton Nuclear Magnetic Resonance
REM: Rapid eye movement
RER1: Retention in Endoplasmic Reticulum 1
SAMP: Senescence-accelerated mouse prone
scRNA-seq.: Single-cell RNA-sequencing
SDS: Sodium Dodecyl Sulfate
SYK: Spleen Tyrosine Kinase
tTa: Tetracycline-controlled trans-activator
Tg: Transgenic
TREM2: Triggering Receptor Expressed on Myeloid Cells 2
TMM: Trimmed Mean of M Values
TNF-α: Tumor Necrosis Factor Alpha
US: United States
VLDL: Very low density lipoprotein
WT: Wildtype
