# Supplementary figures and images for: Updates on mouse models of Alzheimer’s disease
Source: Mol Neurodegener. 2024 Mar 11;19:23. doi: 10.1186/s13024-024-00712-0 (PMC10926682; doi:10.1186/s13024-024-00712-0)

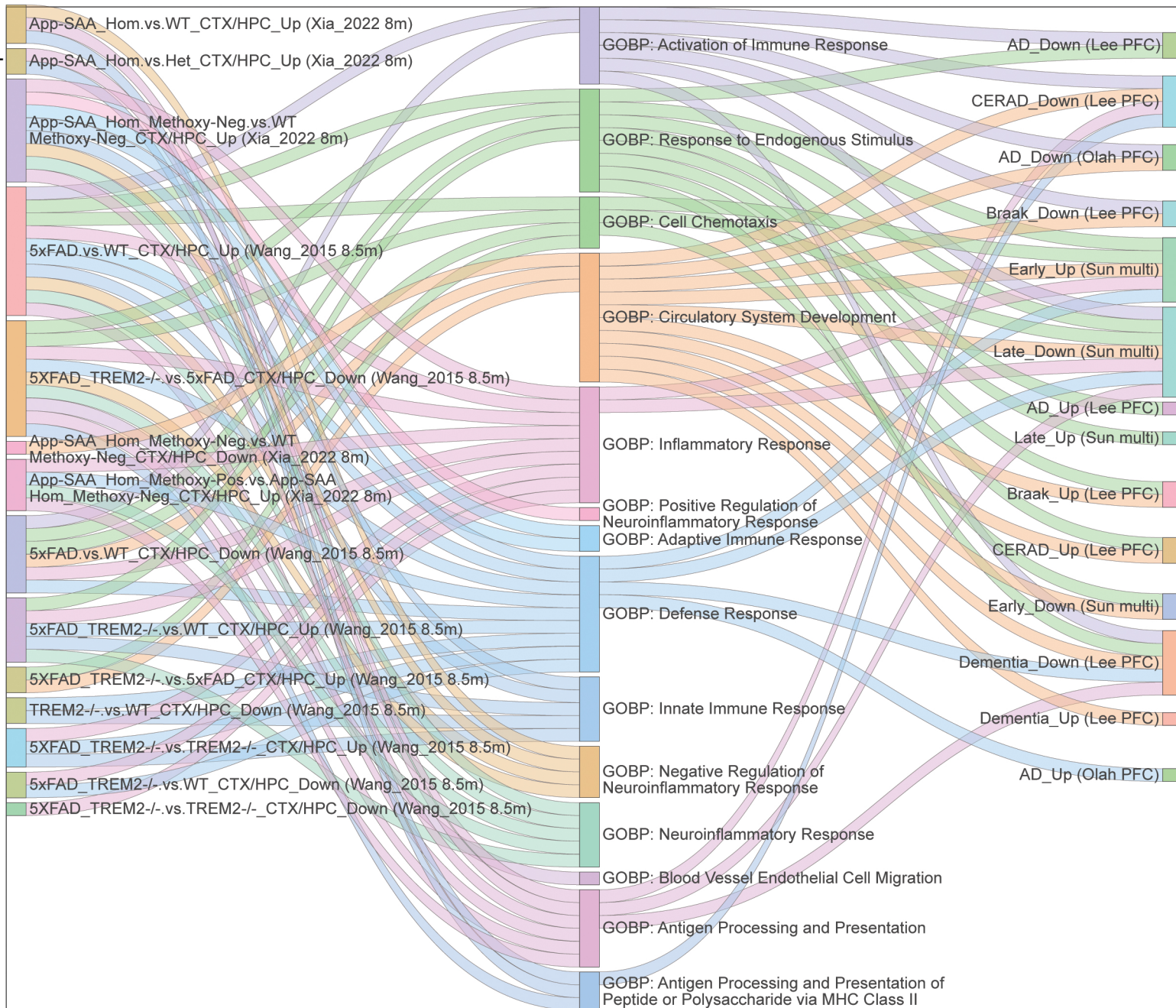

Supplement: Supplementary file 1 — Additional file 1: Supplemental Figure 1. Comparison of gene ontology (GO)/pathways between the AD mouse microglial datasets and human AD microglial datasets. Sanky network plots show the commonly shared GO/pathways between mouse AD gene signatures derived from microglia of AD mouse models (left) and human AD gene signatures derived from scRNA-seq. studies of AD human brain microglia (right). Each node represents a gene signature or a GO/pathway term. Each link colored based of individual GO/pathway term represents a significant overlap between mouse and human gene signatures. Commonly shared GO/pathways involved in neuroinflammation and immune responses in AD. [file 13024_2024_712_MOESM1_ESM.pdf]

Protein, Lipid Metabolism

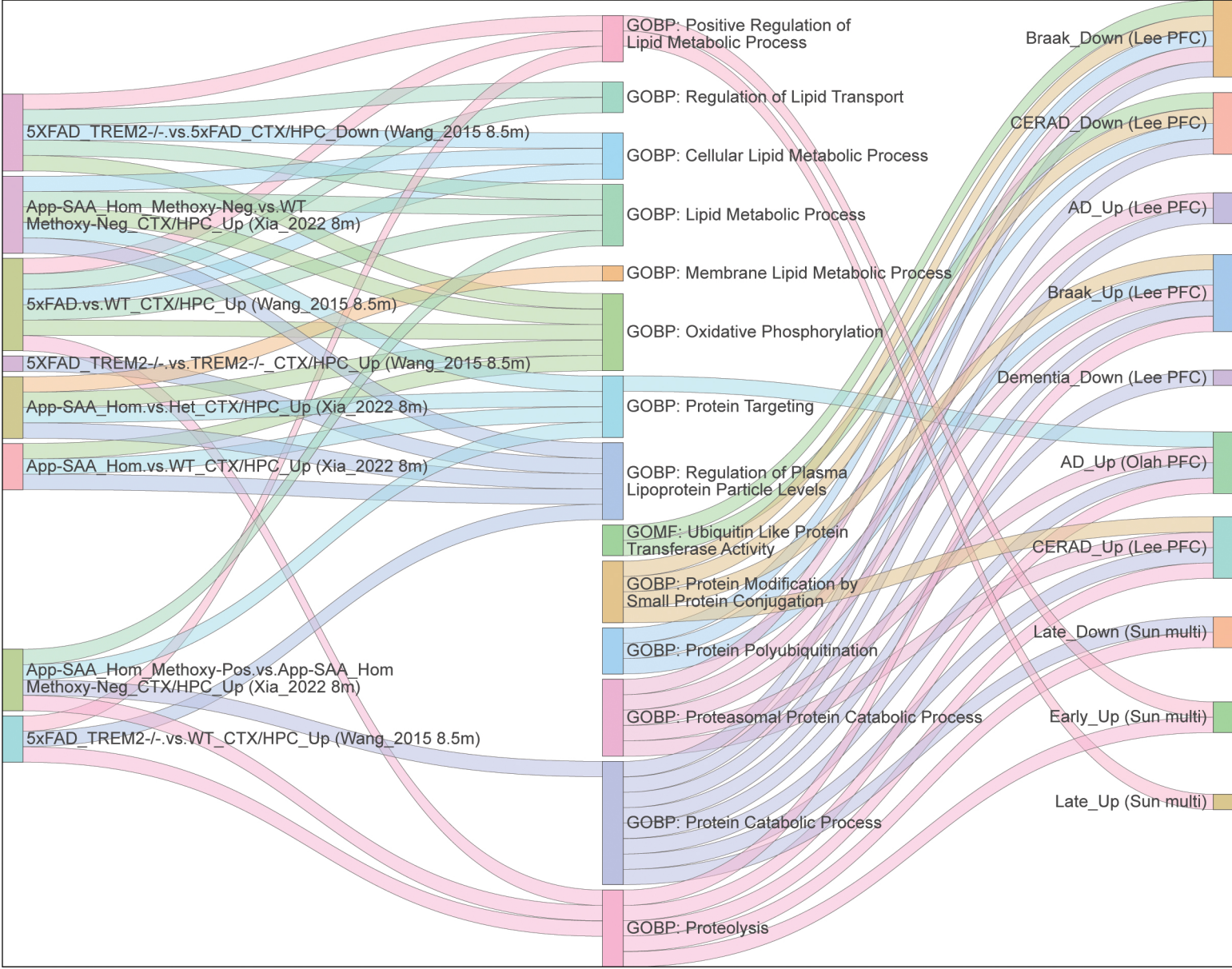

Supplement: Supplementary file 2 — Additional file 2: Supplemental Figure 2. Comparison of gene ontology (GO)/pathways between the AD mouse microglial datasets and human AD microglial datasets. Sanky network plots show the commonly shared GO/pathways between mouse AD gene signatures derived from microglia of AD mouse models (left) and human AD gene signatures derived from scRNA-seq. studies of AD human brain microglia (right). Each node represents a gene signature or a GO/pathway term. Each link colored based of individual GO/pathway term represents a significant overlap between mouse and human gene signatures. Commonly shared GO/pathways involved in protein and lipid metabolism in AD. [file 13024_2024_712_MOESM2_ESM.pdf]
